# The privacy-explainability trade-off: unraveling the impacts of differential privacy and federated learning on attribution methods

**DOI:** 10.3389/frai.2024.1236947

**Published:** 2024-07-03

**Authors:** Saifullah Saifullah, Dominique Mercier, Adriano Lucieri, Andreas Dengel, Sheraz Ahmed

**Affiliations:** ^1^Department of Computer Science, RPTU Kaiserslautern-Landau, Kaiserslautern, Rhineland-Palatinate, Germany; ^2^Smart Data and Knowledge Services (SDS), DFKI GmbH, Kaiserslautern, Rhineland-Palatinate, Germany

**Keywords:** deep learning, explainability, attribution, privacy, federated learning, differential privacy

## Abstract

Since the advent of deep learning (DL), the field has witnessed a continuous stream of innovations. However, the translation of these advancements into practical applications has not kept pace, particularly in safety-critical domains where artificial intelligence (AI) must meet stringent regulatory and ethical standards. This is underscored by the ongoing research in eXplainable AI (XAI) and privacy-preserving machine learning (PPML), which seek to address some limitations associated with these opaque and data-intensive models. Despite brisk research activity in both fields, little attention has been paid to their interaction. This work is the first to thoroughly investigate the effects of privacy-preserving techniques on explanations generated by common XAI methods for DL models. A detailed experimental analysis is conducted to quantify the impact of private training on the explanations provided by DL models, applied to six image datasets and five time series datasets across various domains. The analysis comprises three privacy techniques, nine XAI methods, and seven model architectures. The findings suggest non-negligible changes in explanations through the implementation of privacy measures. Apart from reporting individual effects of PPML on XAI, the paper gives clear recommendations for the choice of techniques in real applications. By unveiling the interdependencies of these pivotal technologies, this research marks an initial step toward resolving the challenges that hinder the deployment of AI in safety-critical settings.

## 1 Introduction

In recent years, a wide variety of deep learning (DL) approaches have achieved outstanding performance in a wide range of application domains (Liu et al., 2019; Sujatha et al., 2021). The versatile applications of deep neural networks in areas such as image processing (Hemanth and Estrela, 2017), object segmentation (Chen et al., 2013), document analysis (Gilani et al., 2017), time series classification (Ismail Fawaz et al., 2019), time series prediction (Lim and Zohren, 2021), layout classification (Binmakhashen and Mahmoud, 2019), sensor analysis, and other areas have contributed to immense growth. Intelligent and automated decision-making bears the potential to improve and transform a row of critical application domains, including finance, healthcare, transportation, and administration. However, DL-based systems rely on complex, data-driven black-box methods whose exact working mechanisms are still widely unexplained in the scientific community (Hassija et al., 2024), while the secure application of algorithms in safety-critical domains requires transparency and traceability of decisions (Khalid et al., 2023). Furthermore, data security is a serious concern in domains involving critical and personal data. DL methods are data-driven, often requiring the transmission, processing, and storage of large amounts of data in multiple remote locations. Various works showed, that even after training, neural networks can leak sensitive information about training data (Fredrikson et al., 2015; Chen and Campbell, 2021). The lack of explainability and privacy of modern DL systems are some of the main challenges that prevent the practical use of these powerful methods in safety-critical domains (Maple et al., 2023; Shaik et al., 2023; Velev and Zlateva, 2023).

The field of eXplainable AI (XAI) seeks to unveil the decision-making processes of black-box models and has been thoroughly researched recently (Das and Rad, 2020; Vilone and Longo, 2020; Hassija et al., 2024). Depending on the area of application, different methods have been developed that attempt to explain the prediction of networks as well as their underlying decision-making process (Simonyan et al., 2013; Zeiler and Fergus, 2013; Springenberg et al., 2014; Ribeiro et al., 2016; Shrikumar et al., 2016, 2017; Lundberg and Lee, 2017; Sundararajan et al., 2017; Zhang and Zhu, 2018). Especially in image processing, so-called attribution methods are commonly used (Simonyan et al., 2013; Zeiler and Fergus, 2013; Sundararajan et al., 2017; Nielsen et al., 2021). These methods generate heatmaps that highlight the areas of the input that were significantly involved in the network prediction.

Some properties of DL-based models (i.e., gradients) are of great importance for decision explanation (Simonyan et al., 2013; Springenberg et al., 2014; Shrikumar et al., 2016, 2017; Sundararajan et al., 2017). However, these properties also provide interfaces for the targeted retrieval of sensitive information. It has been shown that only limited model access suffices to completely reconstruct models and steal their training data (Fredrikson et al., 2015). Moreover, providing additional explanations has been shown to even increase the vulnerability of models in some cases (Shokri et al., 2021). This constitutes a major risk for the deployment of AI in safety-critical applications. Moreover, it is a significant threat to individuals contributing to a model's training data and users alike (Liu et al., 2020). To prevent this, many methods have been designed to protect neural networks from attacks during the training process and to reduce data leakage during later deployment (Abadi et al., 2016; Konečnỳ et al., 2016; Aono et al., 2017; Liu et al., 2020). These methods also open new opportunities for collaboration between multiple parties, allowing for better predictions based on larger amounts of data (Konečnỳ et al., 2016; Mercier et al., 2021).

While XAI methods aim to increase the transparency and intelligibility of a model's decision-making behavior (Hassija et al., 2024), privacy-protection techniques aim to prevent the leakage of sensitive information (Liu et al., 2020). However, the safe deployment of data-driven systems in safety-critical areas is only possible if one can reconcile both goals. To the best of our knowledge, there has been no work that investigates and describes the qualitative and quantitative impact of different privacy-preserving methods on the quality of explanations on a wide range of different deep learning models. Understanding the trade-off between both concurring objectives is crucial to improve XAI methods and assure their correct interpretation in a privacy-preserving setting, constituting an important step in the practical applicability of DL.

**Figure 1:**
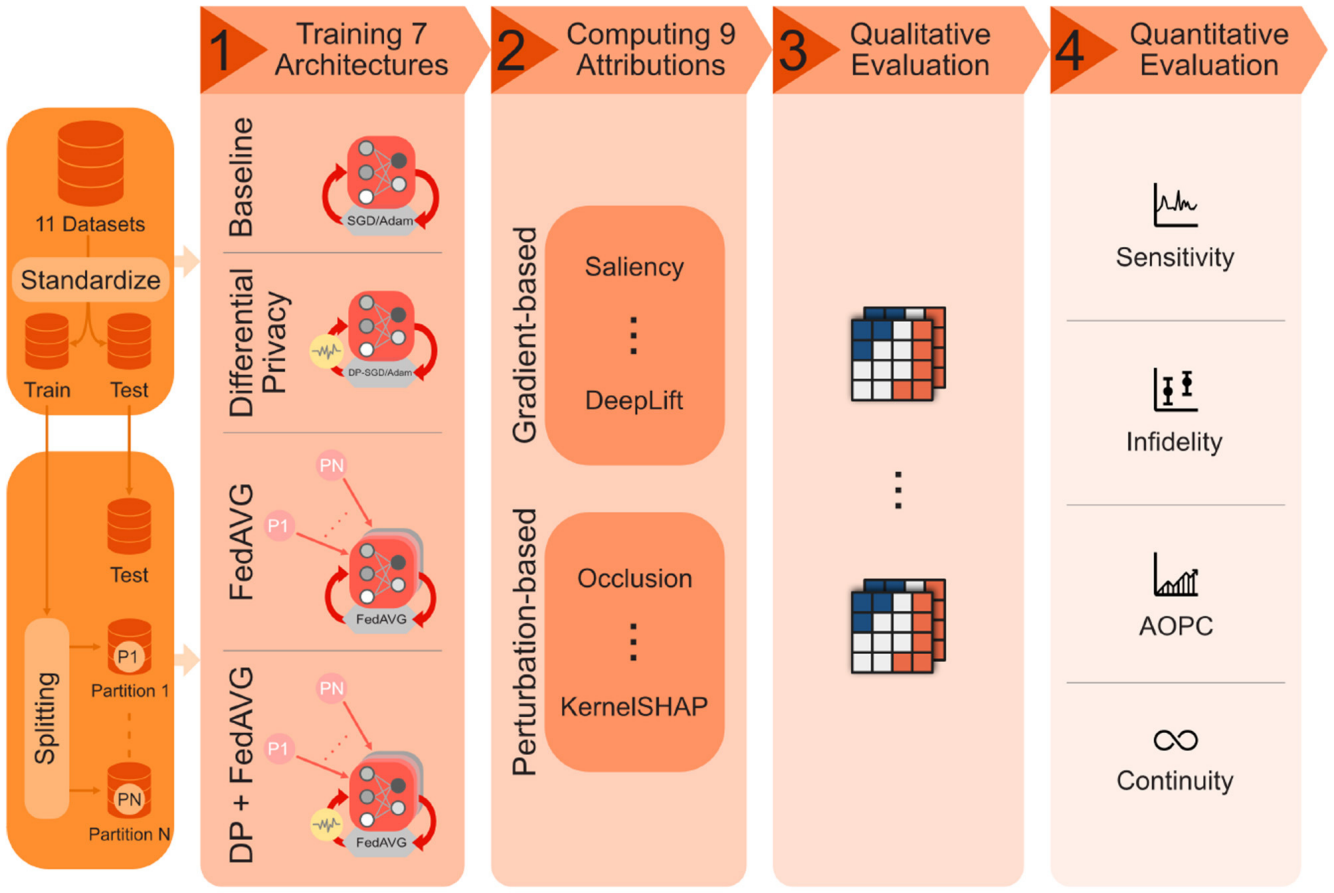
Overview of the conducted analysis on the impact of privacy methods on explainability. The 11 datasets, consisting of time-series and image data from various domains, are first standardized and then split into training and test portions, as well as further training subsets representing different distributed data partitions. The datasets are used to independently train seven model architectures in a non-private baseline as well as three private training settings. Nine different XAI methods are then applied to generate explanations for the resulting 128 models, and qualitatively and quantitatively evaluated using different measures.

This work is the first to thoroughly analyze the influence of privacy-preserving machine learning (PPML) techniques on the explanations generated by XAI methods. In an extensive, multivariate analysis, three different privacy techniques [*Differential Privacy* (*DP*) (Abadi et al., 2016), *Federated Learning* (*FedAVG*) (Konečnỳ et al., 2016), and *Differential Private Federated Learning* (*FedAVG-DP*) (Mercier et al., 2021)] are combined with nine different XAI attribution methods, and applied to seven different model architectures trained on 11 different datasets from various domains including document image, natural image, medical image, as well as time series analysis. The evaluation qualitatively and quantitatively highlights the varying but non-negligible impact of PPML methods on the quality of explanations. An overview of the extensive analysis conducted in the scope of this work is presented in Figure 1. The conducted analysis reveals several important relationships between private training and XAI:

- *Differential Privacy* hampers the interpretability of explanations.
- *Federated Learning* often facilitates the interpretation of generated explanations.
- The *Fidelity* of explanations is potentially deteriorated when using *DP*.
- The negative effects introduced by *DP* can be moderated by combining it with *FedAVG*.
- Perturbation-based XAI methods are less affected by *DP*-based training procedures.

The remainder of this paper is structured as follows. Section 2 gives an overview of relevant XAI methods and PPML techniques. The various datasets used throughout this study are introduced in Section 3. In Section 4 the complete experimental setup is outlined, followed by the presentation of the respective results in Section 5. Trends and findings of this analysis are discussed throughout Section 6 and the manuscript is concluded in Section 7.

## 2 Related work

In the following section, the most common methods in the fields of XAI and PPML will be briefly outlined. A full review of methods is beyond the scope of this work. The interested reader can find extensive reviews of XAI and PPML in Boulemtafes et al. (2020) and Vilone and Longo (2020), respectively.

### 2.1 eXplainable AI

In the field of XAI, attribution methods are very widely used due to their versatility and comprehensibility. Attribution maps approximate the relevance of input features or feature groups to the local model decision and belong to the group of so-called *post-hoc methods* (Fan et al., 2021), which are mainly characterized by their ability to explain models that have already been trained. In 2013, *Saliency* (Simonyan et al., 2013) was published as one of the first methods in this field based on the backpropagation (LeCun et al., 2015) algorithm used to train networks. An extension of this method is *InputXGradient* (Shrikumar et al., 2016), in which the coherence of input features is additionally considered. Other methods that work similarly to the aforementioned methods include *GuidedBackpropagation* (Springenberg et al., 2014) and *IntegratedGradients* (Sundararajan et al., 2017). All these methods are so-called *gradient-based methods* and need access to the networks' internals to compute explanations.

In contrast, *perturbation-based* methods are usually *model-agnostic*, therefore not requiring any specific model architecture to work on. The *Occlusion* (Zeiler and Fergus, 2013) method removes input areas sequentially and reevaluates each manipulated input to measure the influence of single regions on the network prediction. Another subgroup of *perturbation-based* algorithms derives surrogate models from the local model behavior. *Local Interpretable Model-Agnostic Explanations* (*LIME*) (Ribeiro et al., 2016), for instance, applies perturbations to an input sample to obtain a local linear model from these inputs and the respective model predictions. *Shapley Additive Explanations* (*SHAP*) (Lundberg and Lee, 2017) has been proposed as a related method, with additional constraints based on game theory to provide certain mathematical guarantees.

For the sake of completeness, it should be mentioned that there are several other approaches besides the discussed *post-hoc* attribution methods. *Prototype-based* (Li et al., 2018), *patch-based* (Chen et al., 2019), and *concept-based* methods (Kim et al., 2018), for instance, have also been used previously.

### 2.2 Privacy-preserving machine learning

Different techniques have been developed to protect data and model privacy during the training and in the subsequent inference phase. Anonymisation techniques [e.g., K-anonymity (Hellani et al., 2015)] were among the first approaches developed to ensure privacy in model training. Meanwhile, there have been outstanding breakthroughs in the area of privacy attacks. Membership (Rahman et al., 2018) or model inversion attacks (Fredrikson et al., 2015) allow reconstructing training data with extremely limited access to the models. Therefore, simplistic techniques such as anonymization are no longer sufficient.

A promising training technique that leads to a high degree of privacy for data and model is *Homomorphic Encryption* (Aono et al., 2017). However, this method is rarely applicable to modern DL-based systems, due to its massive computational overhead. Another, more frequently used technique is *Differential Privacy* (*DP*) (Abadi et al., 2016). Here, a certain amount of noise is added to the training signal of deep networks to prevent its parameters to capture information held by specific training samples but instead focus on the general characteristics of the whole population. One advantage of this method is that it can be applied to a wide variety of architectures and requires only minimal changes to the training setup. Another prominent technique used to account for data-privacy is *Federated Learning* (*FedAVG*) (Konečnỳ et al., 2016). In *FedAVG*, local models are trained on a data-owner's subset of training samples, and only the locally computed gradients are sent to a centralized server. There, the average is calculated to obtain a global model. This way, sensitive data does not need to leave the institution, but multiple institutions can collaborate to leverage a bigger training set for the global model. Moreover, *FedAVG* can be combined with *DP* to prevent the risk of data leakage from model gradients. Out of the many other privacy techniques (Liu et al., 2020), *DP* and *FedAVG* stand out as the most commonly used.

Several attempts have been made at combining explanations and privacy preservation (Franco et al., 2021; Rahman et al., 2021; Bárcena et al., 2022; Ariffin et al., 2023). Some works investigated the impact of XAI on privacy and found that the privacy of models can indeed be compromised, depending on the XAI method used (Zhao et al., 2021; Goethals et al., 2023; Lucieri et al., 2023; Spartalis et al., 2023; Yan et al., 2023). As a result, methods defending the privacy of explainable models have been proposed (Montenegro et al., 2021; Nguyen et al., 2023; Pentyala et al., 2023). The effect of privacy-preserving training methods on explanations is far less studied (Naidu et al., 2021; Patel et al., 2022; Bozorgpanah and Torra, 2024). This present study tackles the lack of work investigating the overall influence of private training on feature-based explanations in deep learning for different data modalities.

## 3 Datasets

To comprehensively analyze the impact of privacy-preserving methods on explanations, various datasets from different domains in time series and image analysis were utilized, as listed in Table 1.

**Table 1:** Datasets used to evaluate the impact of privacy-preserving training techniques on attribution methods.

**Modality and dataset**	**Domain**	**Train**	**Test**	**Dimensions**	**Channels**	**Classes**
**Time series**						
Anomaly detection	Synthetic	50,000	10,000	50	3	2
Character trajectories	Communication	1,422	1,436	182	3	20
ECG5000	Medical	500	4,500	140	1	5
FordA	Manufacturing	3,601	1,320	500	1	2
Wafer	Information	1,000	6,164	152	1	2
**Images**						
RAF-Database	Facial expressions recognition	12,271	3,068	224^2^	3	7
Caltech-256	Natural image classification	24,485	6,122	224^2^	3	256
ISIC	Medical image analysis	26,521	2,947	224^2^	3	8
SCDB	Synthetic	6,000	1,500	224^2^	3	2
RVL-CDIP	Document analysis	3,20,000	40,000	384^2^/224^2^	3	16
Tobacco3482	Document analysis	2,782	700	384^2^/224^2^	3	10

The datasets cover the tasks of natural image, document image, medical image, and time series classification.

### 3.1 Time series datasets

The first modality that is evaluated is time series data. Time series data is usually acquired using some type of sensor and differs from image data in various characteristics, such as locality constraints and their dependence on a sequential order.

Except for the *Anomaly Detection* dataset (Siddiqui et al., 2019), the datasets for the time series analysis come from the UEA & UCR repository.^1^ This selection includes both univariate and multivariate time series with different numbers of classes, namely *Character Trajectories* (Williams et al., 2006), *ECG5000* (Goldberger et al., 2000), *FordA* (Bagnall et al., 2017), and *Wafer* (Olszewski, 2001). The *Anomaly Detection* dataset and the *FordA* dataset consider the task of anomaly detection. The *Anomaly Detection* dataset deals with point anomalies and the *FordA* dataset with sequence anomalies. Point anomalies are very interpretable for humans, as in their case the data is more or less noise and contains a large peak that indicates the anomaly. Even without the annotation, it is possible to understand whether the explanation for such a sample is correct or not. This is not the case for the *FordA* data, as the sequences are very long and there is no annotation. In this dataset, the anomaly can be a long part of the sequence that varies from the expected behavior. The *Character Trajectories* dataset was selected as it is possible to transform it back to the 2D input space to understand the explanation. It consists of three channels covering the acceleration within the *x* and *y* direction and the pen force. Therefore, it is a real-world dataset that enables precise identification of whether an explanation is good or not. In addition, it is important to mention that the dataset size of the time series datasets differs significantly, to properly represent the influence of data volume.

### 3.2 Image datasets

Image datasets range from specialized domains such as facial recognition, medical image analysis, and document analysis to toy datasets covering a varying number of classes, channels, and dataset sizes.

#### 3.2.1 Natural image datasets

The Real-world Affective Faces Database (*RAF-Database*) (Li and Deng, 2019) is a collection of 15,339 face images with crowdsourced annotations. It classifies images in one of seven facial expressions (Surprise, Fear, Disgust, Happiness, Sadness, Anger, Neutral). *Caltech-256* (Griffin et al., 2007) is a natural image classification dataset comprising a total of 30,607 labeled images with 256 unique object classes.

#### 3.2.2 Medical image datasets

The International Skin Imaging Collaboration (ISIC) provides a large publicly accessible library of digital skin images^2^ and hosts annual challenges. The *ISIC* dataset used in this work is a cleaned combination of all ISIC challenge datasets. The datasets have been merged and freed from duplicates according to the recommendations in Cassidy et al. (2022). All images are labeled as either Melanoma (*MEL*), Nevus (*NV*), Basal Cell Carcinoma (*BCC*), Actinic Keratosis (*AK*), Benign Keratotic Lesion (*BKL*), Dermatofibroma (*DF*), Vascular Lesion (*VASC*), or Squamous Cell Carcinoma (*SCC*). The classification is based on complex combinations of distributed and overlapping biomarkers, posing particular challenges for the explanation of automated decisions. The seven-point checklist criteria dataset (*Derm7pt*) proposed in Kawahara et al. (2019) consists of clinical and dermoscopic images of 1.011 skin lesions with extensive annotation. In this work, only the subset of dermoscopic images along with the respective diagnosis annotations for pre-training of ISIC classifiers is considered in experiments involving *DP*. The *SCDB* (Lucieri et al., 2020) dataset is a synthetic toy dataset inspired by the problems of skin lesion analysis. Images are classified into one of two classes based on the combinations of shapes present in a base shape, depicting the skin lesion. The shapes can be overlapping and redundant, but classification evidence is sparse and localized. Along with the class label, each image is supplemented by shape annotation maps, serving as ground truth explanations.

#### 3.2.3 Document image datasets

Business documents are a fundamental component of modern industry. Recent advances in deep learning have sparked a growing interest in automating document processing tasks such as document search, and extraction of document information. However, business documents often contain highly personal user data and sensitive information pertaining to a company's intellectual property, which makes the secure application of Deep Learning in this area a major concern. On the other hand, deep learning-based decision-making processes have been shown to be susceptible to learning biases in the data (Ntoutsi et al., 2020). An example of such a system involves automatically analyzing resumes to make hiring decisions, which may lead to discrimination against women or members of minority groups. Explainability of such systems is therefore of paramount importance for their safe and practical deployment.

To analyze the interdependence between PPML and XAI for document domain, two popular document benchmark datasets are utilized in this study. *RVL-CDIP* (Harley et al., 2015) is a large-scale document dataset that has been extensively used as a benchmark for document analysis tasks. The dataset contains a total of 400,000 labeled document images with 16 different categories and consists of training, testing, and validation split of 320,000, 40,000, and 40,000 images respectively. *Tobacco3482*,^3^ is another popular but small-scale dataset with 3,482 labeled document images. Since there is no split defined for this dataset, training, testing, and validation splits of sizes 2,504, 700, and 278 images were defined. Since both of these datasets are subsets of a bigger dataset, there exists some overlap between them. Therefore, for all the experiments, the overlapping images were removed from the *RVL-CDIP* dataset. The two datasets were used in combination to analyze the effects of transfer learning on the privacy and explainability aspects of the models.

## 4 Experimental setup

A broad experimental basis, covering various domains, applications, and configurations is necessary, to make general statements about the impact of privacy techniques on explanations. Therefore, a selection of state-of-the-art classifiers is trained on a range of different datasets and applications covering both time series and image domains. Each combination of model and dataset is trained in four different settings, including training without privacy (*Baseline*), with differential privacy (*DP*), federated training (*FedAVG*) and federated training with client-side differential privacy (*FedAVG-DP*). Different explanation methods are finally applied to every model instance to compare their generated explanations.

The subsequent sections provide details about the PPML methods, the XAI methods and their evaluation metrics, as well as the various deep learning models investigated in this work.

### 4.1 PPML methods

For practical reasons, the impact analysis in this work is limited to *Differential Privacy (DP) (Abadi et al.*, *2016**), Federated Learning (FedAVG) (Konečnỳ et al.*, *2016**)*, and the combination of both techniques (referred to as *Differential Private Federated Learning (FedAVG-DP) (Mercier et al.*, *2021**)*.

#### 4.1.1 Differential privacy

*Differential Privacy (DP)* (Dwork, 2006) is a generalized framework for minimizing information release from any given randomized algorithm, and by definition, provides strong protection against several types of privacy attacks, such as, membership inference (Shokri et al., 2017) and linkage attacks (Al-Rubaie and Chang, 2019). Formally, *DP* can be defined as follows:

Definition 1. *Let* M:D↦R *be a randomized algorithm with domain* D *and range* R. M *satisfies* (ε, δ)*-Differential Privacy if for all subsets* S⊆R *and for all datasets* $D,D^{\prime }\in D$ *that differ only in one record*, as can be seen in Equation 1:


(1) $$\begin{array}{l} Pr\left[M\left(D\right)\in S\right]\leq e^{\varepsilon }Pr\left[M\left(D^{\prime }\right)\in S\right]+\delta \end{array}$$

In other words, the output of the algorithm M on any two adjacent datasets $D,D^{\prime }\in D$ should be indistinguishable with high probability, up to a factor of *e*^ε^ and a delta δ. The lower the value of ε, the lower the risk of private information being learned by the models. Note that the definition of *DP* provided above is general and applicable to any given task involving a randomized algorithm M and a dataset *D*, and therefore the definition of dataset adjacency may vary across tasks. Since this work primarily focuses on the classification task involving paired data-label samples, the two datasets are considered adjacent in this work if they differ in a single data-label sample.

##### 4.1.1.1 DP-SGD/Adam

DP-SGD (Abadi et al., 2016) is the primary algorithm when it comes to training deep neural networks under global (ε, δ)-DP. The main working principle of the algorithm is to clip the per-sample model gradients to a fixed bound *C* and then add noise $n\;\sim N\left(0,\sigma^{2}C^{2}\right)$ to them before the parameter updates, thus preventing the leakage of information from each sample. Here, the term σ denotes the noise scale that determines the overall privacy strength, with higher values of σ resulting in stronger privacy constraints (lower ε). In this work, the value of σ is approximated for a given target privacy budget (ε, δ) using the Renyí DP (RDP) privacy accountant (Abadi et al., 2016) that tracks the privacy loss ε given the number of noisy update steps, and a given data sampling rate $q\;\approx \frac{B}{||D||}$, where *B* is the training batch size and *D* is the training dataset. On the other hand, δ is selected as $\delta =\frac{1}{||D||}$ to ensure sufficient privacy. It is also worth mentioning that DP-SGD (Abadi et al., 2016) necessitates the use of Poisson sampling during training (in contrast to the standard random sampling in a non-private setting), which ensures that each training example is sampled with a fixed sampling probability of *q*. Note that DP-SGD can be easily extended to DP-Adam simply by substituting the update step with the update step of the standard Adam optimizer (Kingma and Ba, 2014). This work utilizes both DP-SGD and DP-Adam algorithms for training the models under (ε, δ)-*Differential Privacy*, the complete pseudocodes of which are provided in the Supplementary Appendix.

#### 4.1.2 Federated Learning

*Federated Learning* refers to a class of privacy algorithms that involve training a model on distributed data without requiring the data to be centralized. This work investigates the most popular widely used federated learning approach, namely, *FedAVG*. Given a total of *n*_*c*_ participants involved in the federated training process, in each training round of the *FedAVG* algorithm, a fraction *f* of clients is sampled, which train a local model instance on their own, private data partition for a total of *E*_*local*_ local training epochs. The local model parameters are subsequently sent to a central server after each round, which aggregates the parameters to create a global model. The global model is then sent back to each client to continue the local training in the next round. In this work, the federated setting is simulated by partitioning each original dataset into its *n*_*c*_ homogeneous subsets *D*_*local*_, such that, $||D_{local}||=\frac{||D||}{n_{c}}$. The full pseudocode for the FedAVG algorithm investigated in this work is provided in the Supplementary Appendix.

#### 4.1.3 Differentially private federated learning

Following the work of Mercier et al. (2021), this study additionally investigates the *FedAVG-DP* privacy method, which combines the two approaches, *Differential Privacy (DP)* and *Federated Learning*, by training differentially private models within the *FedAVG* framework. In particular, similar to the *FedAVG, FedAVG-DP* utilizes a distributed training setup where a fraction *f* of clients is sampled in each round to train a local model. However, in each training round, each client trains the local model instances in a private manner for *E*_*local*_ training epochs using the DP-SGD/Adam algorithm instead of following the standard training process. Note that in this case, the local sampling rate for each DP-SGD/Adam training round is computed as $q\;\approx \frac{B}{||D_{local}||}$. Similarly, the $n\;\sim N\left(0,\sigma^{2}C^{2}\right)$ is independently computed for each individual client for a local target privacy budget (ε, δ), where δ is computed as $\delta =\frac{1}{||D_{local}||}$. The full pseudocode for the FedAVG-DP implementation investigated in this work is provided in the Supplementary Appendix.

### 4.2 XAI methods

Some XAI methods pose specific requirements on the model architecture or training procedure, complicating the application of privacy-protection techniques. Therefore, this work solely focuses on the commonly used *post-hoc* explanations. Different attribution methods vary considerably in their realization and their associated underlying assumptions. Therefore, it was decided to apply a broad range of diverse methods differing in their implementations and theoretical foundations. The work covers a total of nine XAI methods, including gradient-based Saliency (Simonyan et al., 2013), InputXGradient (Shrikumar et al., 2016), GuidedBackpropagation (Springenberg et al., 2014), IntegratedGradients (Sundararajan et al., 2017), DeepSHAP (Lundberg and Lee, 2017), and DeepLift (Shrikumar et al., 2017), but also gradient-free methods such as Occlusion (Zeiler and Fergus, 2013), LIME (Ribeiro et al., 2016) and KernelSHAP (Lundberg and Lee, 2017). The following sections briefly describe the XAI evaluation framework and metrics utilized in this work.

#### 4.2.1 Evaluation framework

Let $D=\left\{\left(x_{1},y_{1}\right),\left(x_{2},y_{2}\right),\ldots ,\left(x_{N},y_{N}\right)\right\}$ denote an input dataset of size *N*, where *x*_*i*_ represents either an individual image sample $x_{i}\in {\mathbb{R}}^{C\times H\times W}$ of size *W*×*H* and *C* channels or an input sequence $x_{i}\in {\mathbb{R}}^{C\times L}$ with length *L* and *C* channels, and $y_{i}\in C$ represents its corresponding class label. Let *f*:*x*↦*y* be a black-box neural network, which takes an input sample *x* and returns its predicted class label ŷ∈C. Then, any given *post-hoc* attribution-based XAI method X[f(x),x,ȳ] takes as input the model *f*(*x*), the input image *x* for which to generate the explanation, and the target class label ȳ∈C, and outputs an explanation matrix (*e* ∈ ℝ^*C*×*H*×*W*^ for images or *e* ∈ ℝ^*C*×*L*^ for sequences), which highlights the importance of each feature (pixel or sequence element) in the input regarding the target label ȳ. All the XAI methods investigated in this study utilize the above framework, where the target label ȳ for generating the explanations is kept the same as the predicted label ŷ_*i*_ = *f*(*x*_*i*_) for each input sample *x*_*i*_.

#### 4.2.2 Evaluation metrics

Evaluating explanations and judging their quality is a common problem not only in XAI research (Zhou et al., 2021), but also in the social sciences (Miller, 2019). Multiple evaluation dimensions have to be considered to make clear statements about the impact of privacy-preserving model training on the explainability of DL-based models. Human-centered evaluation is laborious and requires domain experts. Instead, functionality-grounded methods are best suited for the domain- and dataset-wide fair comparison and quality assessment of XAI and are therefore utilized throughout this study.

In the experiments, the focus lies on the two main properties of explanations as defined in Zhou et al. (2021), namely their *Fidelity* and *Interpretability*. *Fidelity* measures soundness and completeness to ensure that explanations accurately reflect a model's decision-making behavior. *Interpretability* refers to the clarity, parsimony, and broadness of explanations, and therefore describes factors related to the ease of communication on the interface of machines and humans. Functionality-grounded methods make use of formal mathematical definitions as proxies of perceived interpretability.

In this work, the *Fidelity* of explanations is quantified using the evaluation metrics *Sensitivity* (Yeh et al., 2019), *Infidelity* (Yeh et al., 2019), *Area Over the Perturbation Curve* (Samek et al., 2016), and *Ground Truth Concordance*.

##### 4.2.2.1 Sensitivity

To measure the *Sensitivity* (Yeh et al., 2019), insignificant perturbations are applied to the input, and the change in the attribution map is measured. Small changes in the input should not result in large changes in the attribution map. Thus, a smaller *Sensitivity* (Yeh et al., 2019) value is better and corresponds to higher fidelity. Formally, the *Sensitivity* S of an explanation generated by a given XAI method X(f(x),x,y) is computed, as can be seen in Equation 2:


(2) $$\begin{array}{l} S=\underset{||x-\hat{x}||\leq r}{\max}||X\left(f\left(x\right),x,ȳ\right)-X\left(f\left(x\right),\hat{x},ȳ\right)|| \end{array}$$

where *f*(*x*), *x*, and ȳ denote the black-box neural network, the input sample and the target class label, respectively, as described in Section 4.2.1, and *r* represents the noise perturbation radius. In practice, *Sensitivity* S is measured by perturbing the input *x* with noise drawn from the uniform distribution ~U(-r,r) for a fixed number of iterations, and the maximum measured value is reported. In this work, *Sensitivity* (Yeh et al., 2019) is evaluated for each XAI method by utilizing a noise perturbation radius of *r* = 0.02 and a total of 50 perturbations per sample.

##### 4.2.2.2 Infidelity

To measure *Infidelity* (Yeh et al., 2019), significant perturbations are applied to both the input and the corresponding attribution map. Then, the mean-squared error is computed between the perturbed attribution map and the difference in the model predictions of perturbed and unperturbed input. Intuitively, the *Infidelity* (Yeh et al., 2019) of an explanation provides direct correlation between the important features determined by the attribution map and their impact on the model output. Formally, the *Infidelity* (Yeh et al., 2019) of an explanation produced by an XAI method X(f(x),x,y) is computed, as can be seen in Equation 3:


(3) $$\begin{array}{l} I={𝔼}_{I\sim N\left(0,\sigma^{2}\right)}\left[{\left(I^{T}X\left(f\left(x\right),x,ȳ\right)-\left(f\left(x\right)-f\left(x-I\right)\right)\right)}^{2}\right] \end{array}$$

where *f*(*x*) is the corresponding black-box neural network, *x* is the input, ȳ is the target class label, and $I\sim N\left(0,\sigma^{2}\right)$ is the noisy baseline which is utilized to perturb the inputs and the attribution maps. In this work, a noise scale σ = 0.003 is used to generate the noisy baseline *I*, with a total of 100 perturbation iterations per sample utilized to compute the *Infidelity* (Yeh et al., 2019).

##### 4.2.2.3 Area over the perturbation curve

The *Area Over the Perturbation Curve* (*AOPC*) (Samek et al., 2016) measures the alignment between an attribution map's relevance values and the effect of perturbing the corresponding input regions on the model prediction. Intuitively, removing features with lower importance should affect the prediction less than the deletion of important features. *AOPC* (Samek et al., 2016) is computed by integrating the model's output confidence scores over the consecutive perturbations with either decreasing (starting from the most relevant features, MoRF) or increasing attribution importance (starting from the least relevant features, LeRF), relative to random perturbations. Therefore, higher values for the MoRF case and lower values for the LeRF case indicate a higher faithfulness of the attribution map. For both scenarios, the *AOPC* metric for an explanation is formally computed, as can be seen in Equation 4:


(4) $$\begin{array}{l} {\text{AOPC}}_{MoRF,LeRF}=\frac{1}{L+1}\frac{1}{N}\overset{N}{\underset{i=1}{\sum }}\overset{L}{\underset{k=1}{\sum }}\hat{f}\left(x_{i,\mathbb{P}}^{0},ȳ\right)-\hat{f}\left(x_{i,\mathbb{P}}^{k},ȳ\right) \end{array}$$

where $\hat{f}\left(x,ȳ\right)$ refers to the output confidence score of the black-box neural network *f*(*x*) for the target label ȳ, *N* is the total number of dataset samples, and *L* is the total number of perturbations applied to the input starting from the most or least relevant features. In this work, the mean RGB perturbation (Samek et al., 2016) is applied for generating the AOPC, where the input image samples are perturbed by replacing square patches of size *p*×*p* with the mean of the dataset. The patch sizes used for the resolutions 224 × 224 and 384 × 384 are 16 × 16 and 24 × 24, respectively, and a total of *L* = 100 feature removal steps are utilized to compute the AOPC metric. In addition, to summarize the AOPC as a scalar value, in this work, the final AOPC scores are generated as the total area between the computed AOPC_MoRF_ and the AOPC_LeRF_ curves.

##### 4.2.2.4 Ground truth concordance

Most fidelity measures evaluate the degree to which an explanation is faithful to the local model behavior. Whether the explanation is human-aligned, on the other hand, can only be evaluated with ground truth explanations available. In this study, *Ground Truth Concordance* is measured by computing the overlap between the input region with the highest attribution and the corresponding segmentation map of the ground truth explanation. First, attribution is aggregated over the channels, and only positive values are considered. All attribution maps are blurred before computing the concordance, to moderate the drawback of gradient-based methods, which generate noisier explanations by design. Binarization is performed in ten equidistant thresholding steps on values normalized in the range [0, 1] to be independent of any particular threshold value. In this study, *Ground Truth Concordance* (*GTC*) is defined as the area of overlap between the binarized attribution map and the ground truth explanation, divided by the whole area of attribution after binarization. This can also be expressed as the ratio of correctly predicted positive observations to the total predicted positives, as can be seen in Equation 5:


(5) $$\begin{array}{l} GTC=\frac{TP}{TP+FP} \end{array}$$

Note that this definition here coincides with that of the standard *Precision* metric, differing in that *GTC* is specific to the spatial comparison of an attribution map with a ground truth segmentation.

##### 4.2.2.5 Continuity

The *Interpretability* of explanations was measured using the *Continuity* metric. In general, humans have difficulties interpreting information that is both high dimensional and scattered. *Continuity* is defined as the sum of the absolute changes between two consecutive importance scores in an attribution map. For time series, the continuity is the absolute change between each subsequent point in a sequence, whereas in the image domain, the absolute changes are measured and aggregated separately in X and Y directions. Better *Interpretability* is indicated by lower continuity scores.

All evaluation metrics were computed on the respective test datasets. The attribution maps were normalized to have zero mean and unit standard deviation for a fair comparison across methods and different privacy types. Due to computational and time restrictions, the influence of PPML on attribution methods was quantified using a subset of the respective test sets, limited to a maximum of 1,000 examples. This work assumes that 1,000 randomly selected examples represent a sufficient quantity to generalize the findings to the complete test datasets.

### 4.3 Critical difference diagrams

Intuitive visualization of high-dimensional data is particularly challenging when the data origins from multiple distinct configurations, as in this case. Critical Difference (CD) diagrams, proposed by Demšar (2006), allow the high-level visualization of complex experimental data intuitively and were therefore chosen to present most quantitative results. Their ability to condense ordinal information across different datasets, models, and attribution methods extracts relevant information and helps to pick up universal trends in a benchmark study. Moreover, the method includes statistical tests, indicating the data's significance.

CD diagrams report the exact average rank of a given item, in a series of different settings. If the statistical significance for two distinct items is not guaranteed, these items are connected by a colored horizontal line and referred to as a “clique.” In this study, the Friedman test is used to decide the statistical significance of a group of different observations. The Holm-adjusted Wilcoxon's signed rank test is then applied for *post-hoc* analysis, as suggested in Benavoli et al. (2016). For all statistical analyses, an α of 0.05 is assumed.

### 4.4 Models

In time series analysis, *InceptionTime* (Ismail Fawaz et al., 2020) and *ResNet-50* (He et al., 2016) were used as representative networks, achieving state-of-the-art performance for the used datasets. *ResNet-50* (He et al., 2016), *NFNet* (Brock et al., 2021) and *ConvNeXt* (Liu et al., 2022) have been used for classification of natural and medical images. Since document images differ significantly from natural images, a different set of models has been used for this domain, including *AlexNet* (Krizhevsky et al., 2017), *VGG-16* (Simonyan and Zisserman, 2014), *ResNet-50* (He et al., 2016), *EfficientNet* (Tan and Le, 2019), and *ConvNeXt* (Liu et al., 2022), which have shown the best performance in the past.

### 4.5 Implementation details

Baseline networks were trained using the standard SGD or ADAM optimizers with varying numbers of epochs per dataset, to ensure convergence. For all other settings, training and privacy hyperparameters have been manually tuned to find a good trade-off between privacy and model performance matching the baseline. This is important to guarantee a sufficiently fair comparison between the methods, since a significantly worse network would also show worse attribution results. However, all models were trained with overall comparable settings. Moreover, fixed seeds were used to ensure reproducibility. The training data was split between training and validation with a factor of 0.9, wherever no validation dataset had been provided. The reported performances correspond to the test accuracies achieved by the models performing best on the validation sets.

## 5 Experimental results

### 5.1 Effect of PPML on model performance

Privacy-preserving training techniques can have a significant effect on the model performances. The severity depends on multiple factors including model architecture, type of dataset, as well as various hyperparameters for model training. Table 2 shows the results on the respective test sets for all experiment configurations when trained with different private training techniques. All results are sorted by domains and datasets to provide a better overview.

**Table 2:** Test accuracies on all datasets for different architectures and privacy-preserving training settings, divided by application domain.

	**Datasets and models**	**Acc_Baseline_**	**Acc_DP_/ϵ**	**Acc_FedAVG_**	**Acc_FedAVG − DP_/ϵ**
**Time series analysis**	**Anomaly**			*n*_*c*_ = 4	*n*_*c*_ = 4
		InceptionTime	98.74	92.87 / 5.0	98.77	89.50 / 5.0
		ResNet-50	98.70	97.02 / 5.0	98.60	97.36 / 5.0
		**Character Traj**.			*n*_*c*_ = 4	*n*_*c*_ = 4
		InceptionTime	99.44	91.85 / 5.0	98.82	87.26 / 50.0
						68.73 / 5.0
		ResNet-50	99.44	85.03 / 5.0	98.19	82.10 / 50.0
						59.19 / 5.0
		**ECG5000**			*n*_*c*_ = 4	*n*_*c*_ = 4
		InceptionTime	94.38	89.07 / 5.0	93.36	89.29 / 5.0
		ResNet-50	94.16	89.64 / 5.0	92.78	88.87 / 5.0
		**FordA**			*n*_*c*_ = 4	*n*_*c*_ = 4
		InceptionTime	95.61	92.88 / 5.0	97.70	94.17 / 50.0
						91.43 / 5.0
		ResNet-50	94.32	86.14 / 5.0	93.94	87.12 / 50.0
						76.44 / 5.0
		**Wafer**			*n*_*c*_ = 4	*n*_*c*_ = 4
		InceptionTime	99.22	89.21 / 5.0	97.81	89.21 / 5.0
		ResNet-50	98.75	89.21 / 5.0	89.21	89.21 / 5.0
**Document analysis**	**RVL-CDIP**			*n*_*c*_ = 8	*n*_*c*_ = 8
		AlexNet	87.90	70.30 / 4.5	85.54	61.35 / 5.3
		VGG-16	91.00	69.67 / 4.4	89.41	62.38 / 5.4
		ResNet-50	90.50	72.55 / 5.0	88.25	68.85 / 8.8
		Efficientnet-B4	92.60	60.20 / 4.2	90.59	45.09 / 6.5
		ConvNeXt-B	93.64	75.60 / 3.7	92.60	73.23 / 7.7
		**Tobacco3482**			*n*_*c*_ = 4	*n*_*c*_ = 4
		AlexNet	89.57	86.14 / 3.9	91.85	85.71 / 8.0
		VGG-16	94.14	85.14 / 4.9	93.99	87.00 / 7.5
		ResNet-50	92.57	75.42 / 2.7	92.14	78.43 / 7.3
		Efficientnet-B4	94.42	89.42 / 4.4	93.99	88.57 / 8.0
		ConvNeXt-B	94.71	87.14 / 4.8	94.85	85.42 / 6.0
**Natural Images**	**Caltech-256**			*n*_*c*_ = 4	*n*_*c*_ = 4
		ResNet-50	87.30	59.00 / 5.0	87.97	61.82 / 35.94
		NFNet	88.50	60.17 / 5.0	91.39	75.28 / 35.13
		ConvNeXt-B	91.57	78.32 / 5.0	93.74	79.86 / 14.85
		**RAF-database**			*n*_*c*_ = 4	*n*_*c*_ = 4
		ResNet-50	81.91	67.42 / 5.0	80.82	64.43 / 13.23
		NFNet	83.96	69.68 / 5.0	82.82	69.75 / 13.33
		ConvNeXt-B	86.63	69.32 / 4.79	88.23	71.97 / 13.23
**Medical**	**ISIC**			*n*_*c*_ = 4	*n*_*c*_ = 4
		ResNet-50	86.08	71.09 / 4.66	82.15	70.89 / 8.09
		NFNet	90.16	77.23 / 14.61	86.90	71.39 / 18.28
		ConvNeXt-B	87.20	69.63 / 14.02	81.87	68.78 / 30.69
**Synthetic**	**SCDB**			***n*_*c*_ = 4**	***n*_*c*_ = 4**
		ResNet-50	90.20	86.20/4.60	92.19	85.13/60.00
		NFNet	94.40	88.33/4.46	94.87	85.19/18.27
		ConvNeXt-B	92.46	85.80/13.19	92.40	87.07/30.08

For configurations containing *DP* or *FedAVG*, the ϵ and *n*_*c*_ values are provided, respectively. A lower ϵ corresponds to stronger privacy. *n*_*c*_ represents the number of clients used to simulate the federated settings.

Even in privacy-preserving training settings, all models converged and demonstrated acceptable accuracies. However, the best accuracies were usually achieved in *Baseline* or *FedAVG* settings. Across all domains, it can be observed that *DP* has a considerable impact on the models' test performances. For some configurations, a higher ϵ-value was required to achieve comparable results (e.g., *NFNet* and *ConvNeXt-B* for *ISIC*). However, no consistent pattern indicating higher robustness of one model architecture over another, against noise introduced by *DP*, is obvious. In contrast to *DP, FedAVG* always resulted in significantly lower performance losses. The combination of *FedAVG* and *DP* almost exclusively resulted in a lower performance, considering a comparable ϵ-value.

The results from the time series domain for most datasets indicate that *InceptionTime* is usually affected slightly less by private training, in direct comparison with *ResNet-50*. The only exception is the *Anomaly* dataset, which experienced almost no performance loss with *ResNet-50*. One possible explanation for this is the advanced architecture of *InceptionTime* including residual connections and inception modules. This enables *InceptionTime* to be more robust against noise and outliers. In the time series domain, the two datasets *FordA* and *Character Trajectories* considerably suffered from the combination of *DP* with *FedAVG*. For these datasets, the ϵ-value had to be increased to achieve adequate results.

The results from the image domain indicate similar findings. Since the models for the *Tobacco3482* dataset were trained after being pretrained on the *RVL-CDIP* dataset, the models were able to achieve higher performance even with *DP* and *FedAVG-DP*. However, despite pretraining on *Derm7pt*, the impact of *DP* on *ISIC*-trained models is still considerable. For all datasets in the image domain, a moderately higher ϵ-value was also used for experimentation, to improve the performance of the models for the *DP* and *FedAVG-DP* cases. However, it did not seem to provide any significant improvement in most of the cases. It is worth noting that for larger datasets (i.e., *RVL-CDIP, Caltech-256, RAF-Database*, and *ISIC*), *DP* and *FedAVG-DP* severely degraded the performance of the models whereas the performance for *FedAVG* is still comparable to the *Baseline* setting.

### 5.2 Qualitative impact analysis of PPML on attribution methods

A visual inspection of individual explanations gives a first impression of the influence privacy-preserving techniques can have on the trained models. These local impressions are then further validated on the dataset level through the qualitative analysis of summary statistics.

For space reasons, we have limited ourselves to presenting results based only on the widely used *ResNet-50* architecture, if not otherwise stated. The fact that this model is readily applicable to both time-series and image domains made it particularly suitable. Moreover, the overall results did not vary too much between the different model architectures. Additional qualitative results are presented in the Supplementary Appendix.

#### 5.2.1 Analysis of individual sample attributions

**Figure 2:**
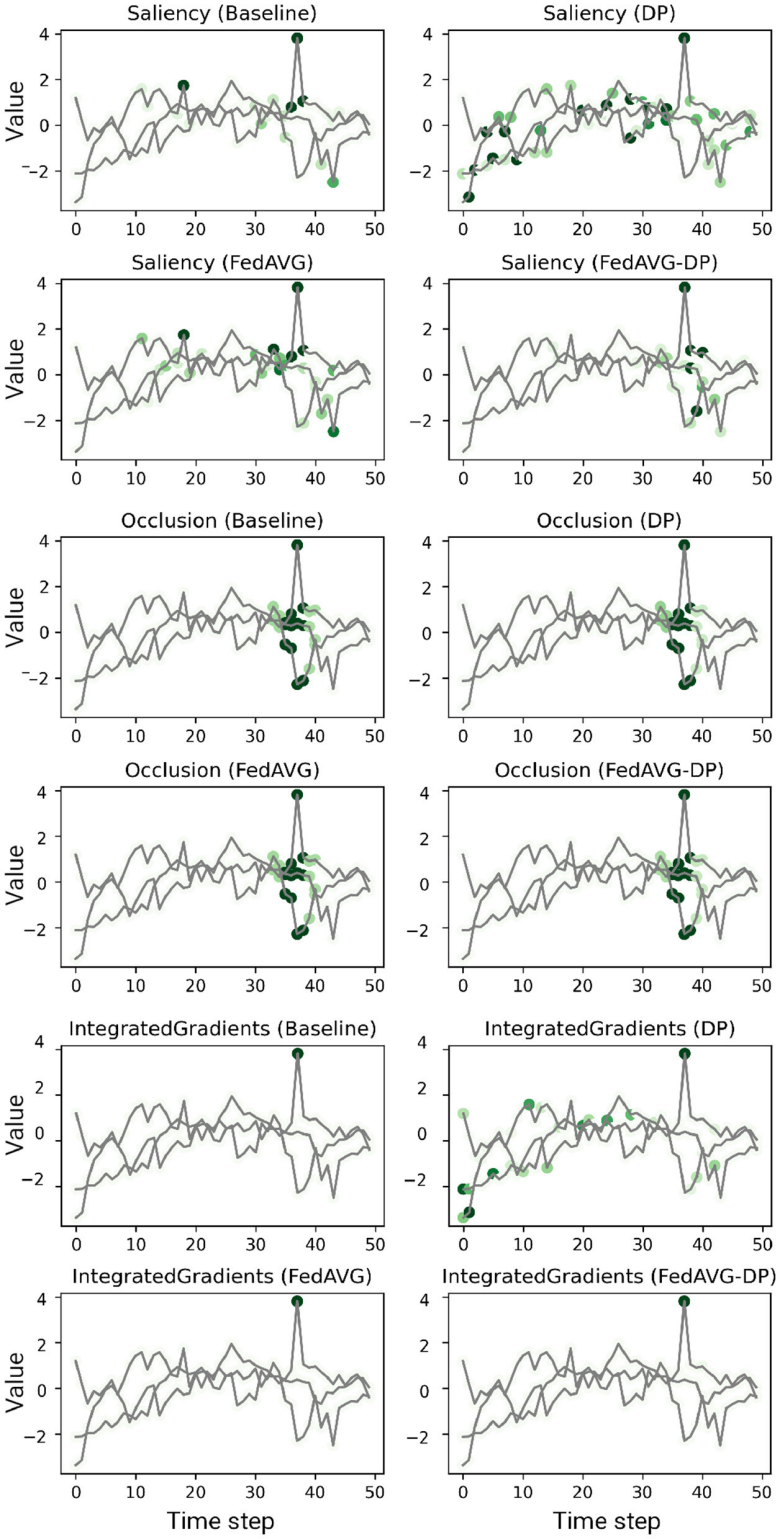
Examples of computed attributions for three samples using a *ResNet-50* trained on the *Anomaly Detection* dataset. Each two consecutive rows show the results for four explanation methods on one individual sample. *DP*-based training techniques tend to add additional noise and alter the explanation. *FedAVG*, by contrast, is closer to the original attribution of the baseline.

**Figure 3:**
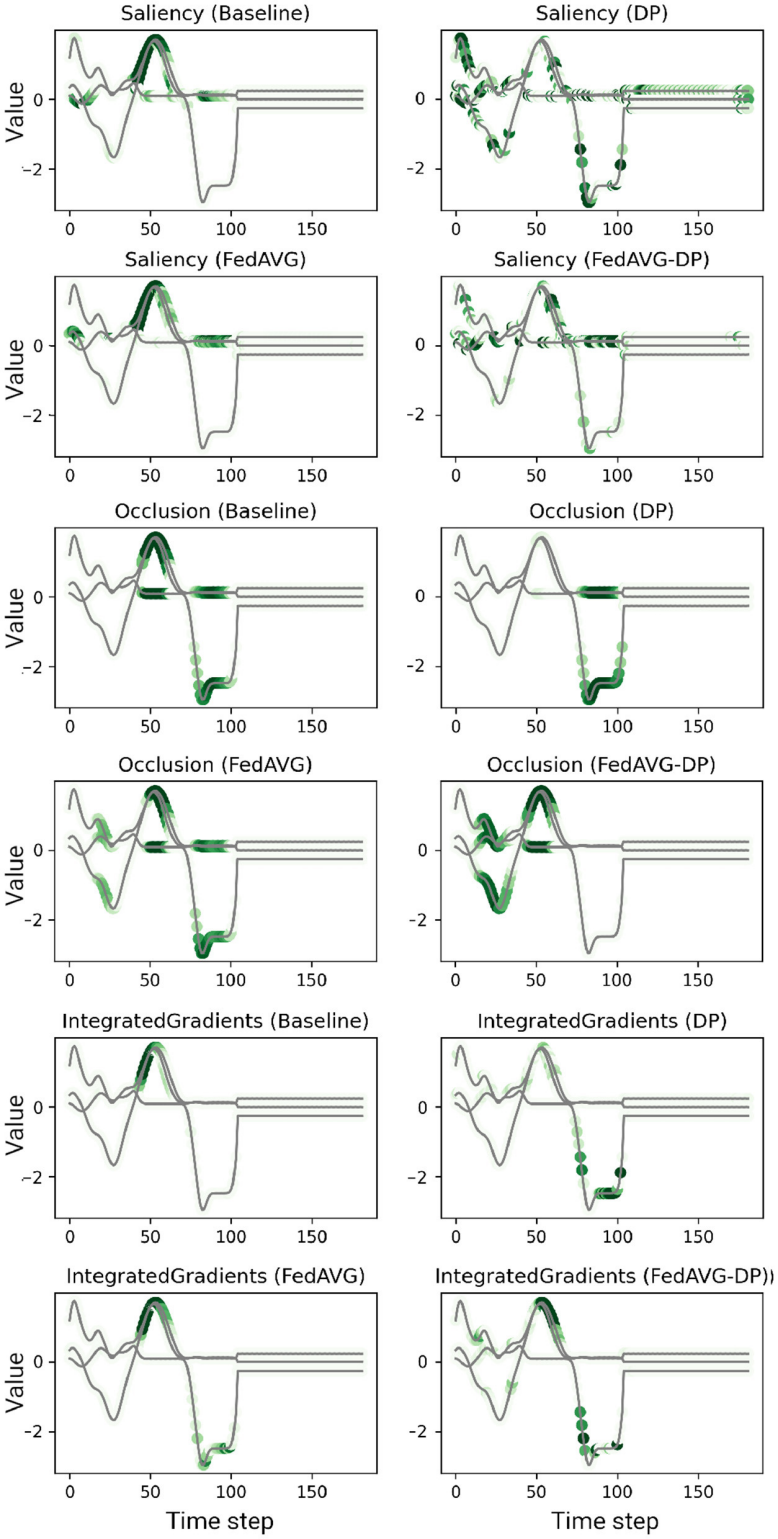
Examples of computed attributions for three samples using a *ResNet-50* trained on the *Character Trajectories* dataset. Each two consecutive rows show the results for four explanation methods on one individual sample. *DP*-based training techniques tend to add additional noise and alter the explanation. *FedAVG*, by contrast, is closer to the original attribution of the baseline.

Figures 2, 3 show explanations generated in the time series domain using the *Anomaly Detection* and *Character Trajectories* datasets, respectively. For the *Anomaly Detection* dataset, it can be observed that there is a general overlap between the explanations from different training settings, always highlighting the anomaly. In some cases, *DP* increases the amount of noise in the signal's relevance around the anomaly, yielding unclear and misleading explanations by highlighting distant points which do not correspond to the anomaly at all. However, this is not the case when additionally adding *FedAVG* in the *FedAVG-DP* setting. By contrast, *DP*-trained models show remarkable deviations from the original *Baseline* explanation when trained on the *Character Trajectories* dataset. This observation holds not only true for *DP* but also *FedAVG-DP* settings. *FedAVG*, on the other hand, shows explanations close to the *Baseline* setting, with only minor deviations.

**Figure 4:**
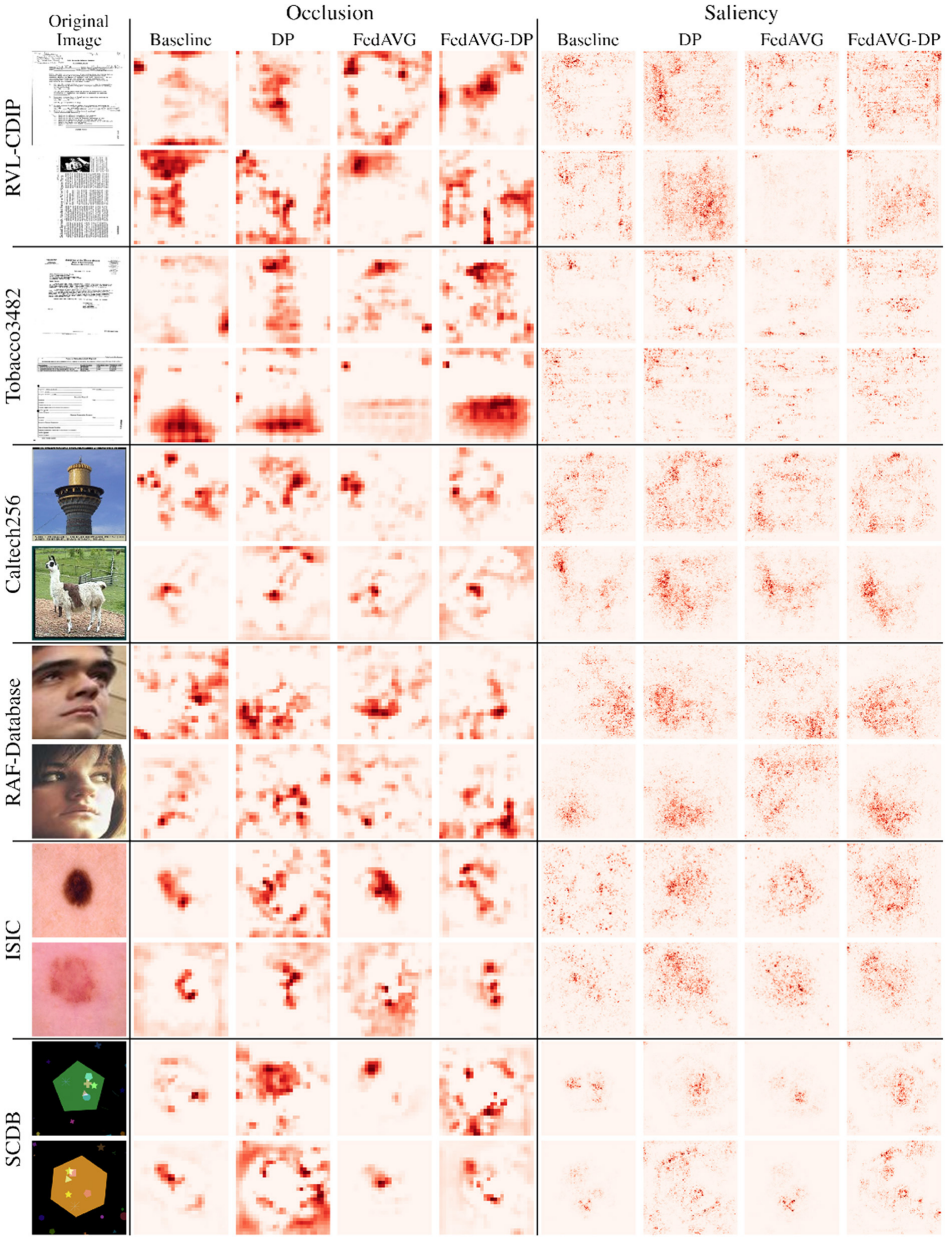
Examples of perturbation- and gradient-based attribution maps computed on models trained in varying privacy configurations. Two random, correctly classified samples are provided per dataset. *Occlusion* and *Saliency* attribution maps are computed on *ResNet-50* models. Red regions indicate positive attribution relevance. Images 5 and 6 are taken from Kaggle (https://www.kaggle.com/datasets/jessicali9530/caltech256). Images 7 and 8 are taken from the RAF-Database (http://www.whdeng.cn/raf/model1.html).

Figure 4 shows samples from all image datasets along with the generated *Occlusion* and *Saliency* explanations from *ResNet-50* models trained with and without privacy techniques. It can be seen that different training settings yield heatmaps that visibly differ. However, the areas of highest relevance roughly overlap for most samples. Particularly for *Saliency* attributions, it can be observed that *DP* and *FedAVG-DP* often add additional noise to the generated explanations. In some cases, this can also be observed in *Occlusion*. This is particularly striking in *SCDB* samples in the last two rows, where both *DP*-based methods highlight regions outside the decision-relevant area. Another striking example is the second sample from *RVL-CDIP*. It can be seen that both *FedAVG* attribution maps present smoother and more focused heatmaps pointing to a specific location on the image. On closer inspection of the samples from document datasets, it was found that *FedAVG* prominently focused on specific class-relevant cues such as dates, titles, figures, etc. Moreover, comparing attribution maps from models trained in the *DP* and *FedAVG-DP* settings, it can be observed that the addition of *FedAVG* leads to less noise in the attribution for some samples.

When observing the *SCDB* sample in the last row, it can be observed that for the *Occlusion* method, the *Baseline* setting highlights both rectangle and star shapes, whereas the model trained with *FedAVG* only focuses on the rectangle shapes. In *SCDB*, rectangles are exclusive markers for class two. However, both star and star markers are also part of the decision-relevant shape combination. *FedAVG* appears to have focused only on the single relevant marker, whereas in the *Baseline* setting, multiple relevant markers were highlighted. This is also evident from the *SCDB* sample in the second last row, where *FedAVG* successfully focused on the ellipse shape, which is exclusive for class one. It has to be noted that the *Occlusion* attribution map shows two relevant regions, where the most relevant region highlights the edge of the big, green pentagon. This could be attributed to a limitation of *Occlusion*, corresponding to distraction due to the generation of out-of-distribution samples during perturbation. Interestingly, *Occlusion* attribution maps for the *Baseline* and *FedAVG* settings show different decision-relevant cues for the last row's sample, as compared to the corresponding *Saliency* maps. The former highlights rectangle and star shapes, whereas the latter most prominently highlights the star marker on the lower part of the image. As already mentioned, star markers are also decision-relevant for that sample. However, they are no exclusive markers and could also indicate shape combinations appearing in class one. Nevertheless, it has to be mentioned that for some individual samples, these observations do not apply. For the second sample of *ISIC* and the first sample of *RAF-Database*, for instance, *Baseline* and *FedAVG* did not yield clearer heatmaps as compared to the *DP*-based approaches. To capture overall trends and characteristics in attribution maps of different configurations, further analysis on dataset-level statistics of the generated attribution maps was performed.

#### 5.2.2 Dataset-wide analysis

**Figure 5:**
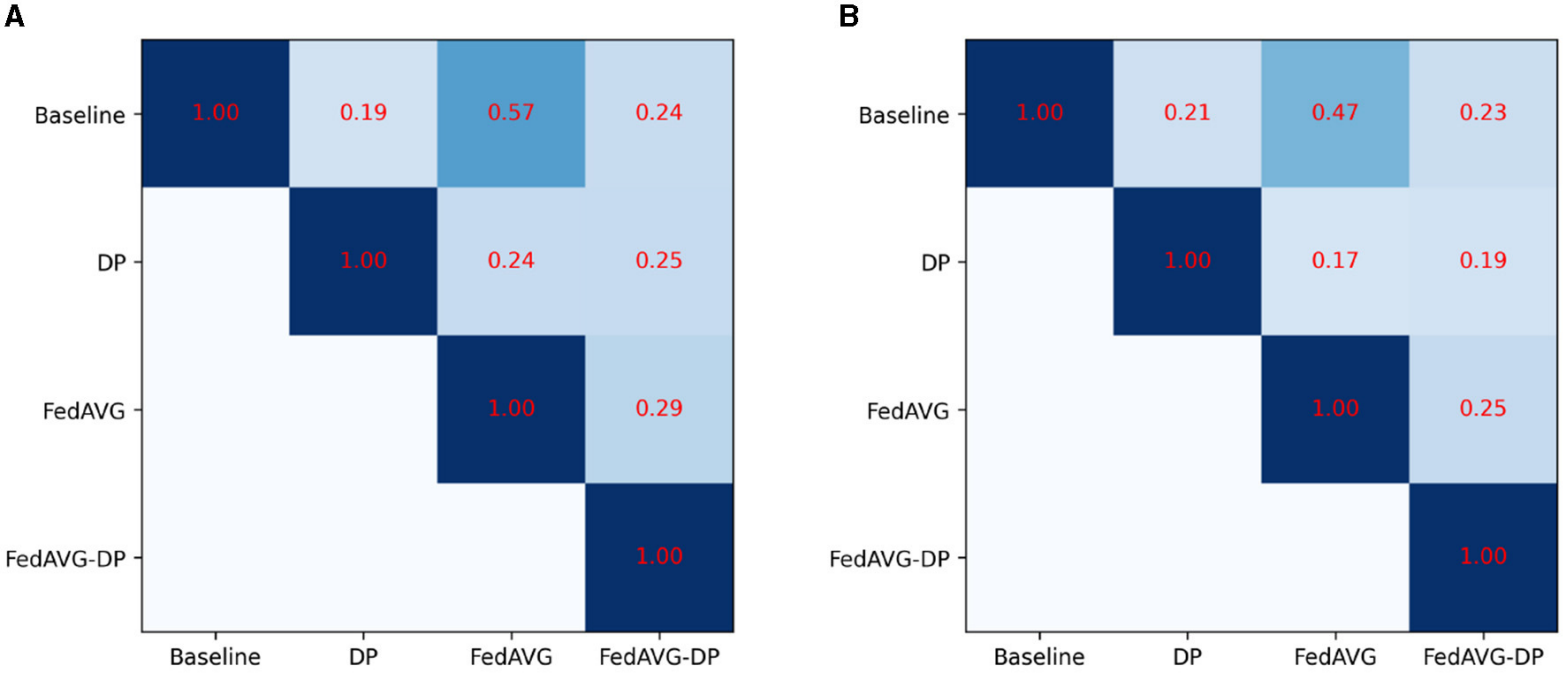
Average Pearson correlation of the attribution maps compared between the different privacy approaches for the *Anomaly Detection* dataset. *FedAVG* shows a higher similarity to the *Baseline* setting, as compared to the *DP*-based approaches. **(A)** InceptionTime. **(B)** ResNet.

Figure 5 shows the Pearson correlation of the explanations generated by different training settings for the *Anomaly Detection* dataset. Therefore, the correlation across the different training approaches was computed using all available attribution maps. Precisely speaking, the correlation between the corresponding attribution maps was calculated and the average over the number of samples was taken. The final correlation shows the score averaged over the attribution methods and the samples. For both architectures, *InceptionTime* and *ResNet-50*, it is evident that the privacy methods significantly change the produced attribution maps. However, *FedAVG* yields significantly higher correlation to the *Baseline* setting as compared to the *DP*-based approaches. Moreover, it is surprising that the correlation between *DP* and *FedAVG-DP* is rather low. The remaining correlation matrices can be found in the Supplementary Appendix, showing similar results.

**Figure 6:**
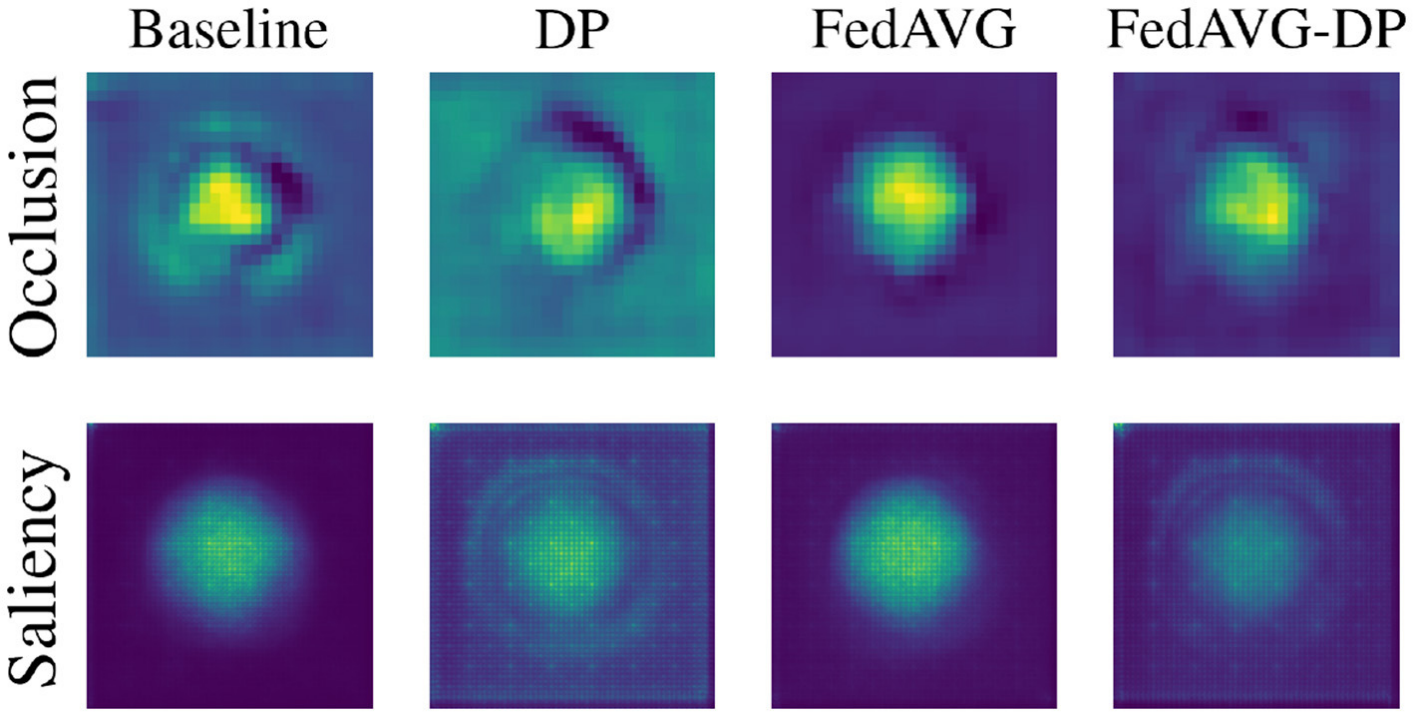
Average attribution heatmaps over all samples and models trained on the *SCDB* dataset in *Baseline, DP, FedAVG*, and *FedAVG-DP* settings.

*SCDB* is a synthetic dataset that, by design, carries all relevant information in the center of the image. Figure 6 shows a visual comparison of the overall impact of privacy on the distribution of attribution in the explanations. For each setting, the attribution maps are averaged across all test samples to highlight the frequency with which regions were attributed as relevant. It is evident that involving *DP* during training drastically increased the diffusion of attribution values, also including image areas that are not related to the actual classification task. Combining *DP* with *FedAVG*, on the other hand, led to a moderation of the noise added by *DP*. Interestingly, the results indicate that *FedAVG* alone usually results in the cleanest and most focused heatmaps, even improving the baseline. As the remaining datasets possess less spatial standardization, it is not trivial to interpret their results in the same way.

This qualitative analysis already drew an interesting initial picture, proving that to some degree, any privacy-preserving training technique has an impact on the generated explanations. Furthermore, the results suggest that *DP*-trained models generate explanations that tend to be noisier and cover potentially unimportant regions, harboring the danger of misleading the explainer. However, it is unclear whether noise added by *DP* only concerns the explanations, or whether this reflects the model decision (fidelity). First results also indicate that the *FedAVG* approach can even improve explanations, leading to more focused, and meaningful explanations in some instances.

### 5.3 Quantitative impact analysis of PPML on attribution methods

The quantitative analysis serves to further verify the findings from the previous section and allows the investigation of whether privacy-preserving techniques impact only the explanations, or also the underlying model behavior.

#### 5.3.1 Continuity

Measuring the continuity of an explanation helps to understand how difficult the interpretation of an explanation might be for an explainer. Humans usually struggle when confronted with high-dimensional, diffuse data.

**Figure 7:**
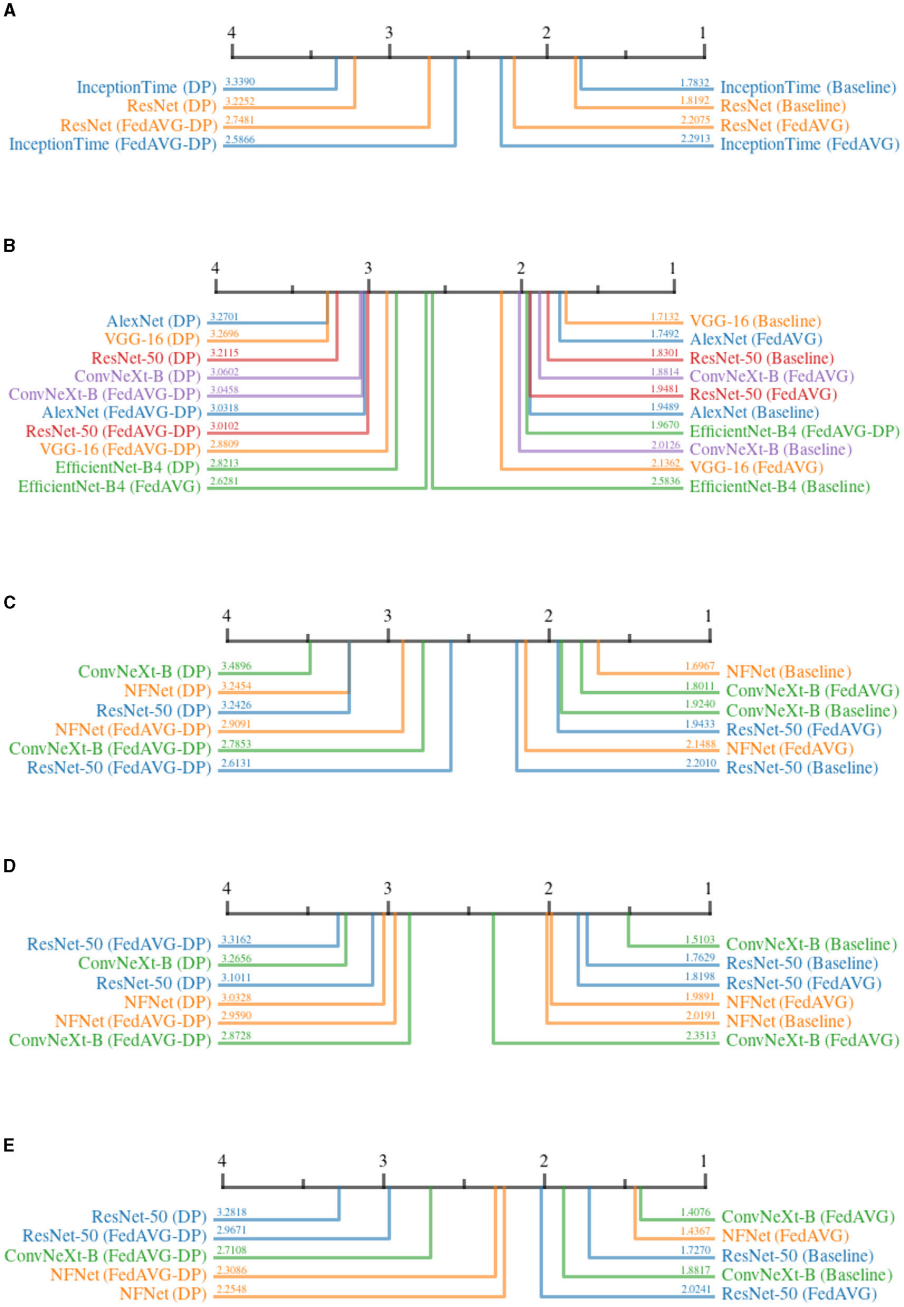
Critical difference diagrams for *Continuity*. The black bar represents the rank of each of the four individual training settings averaged over all datasets for an individual model. Each color represents a single architecture, and the exact rank for a particular combination is provided on the colored line. Privacy results in less continuity and therefore noisier explanations. **(A)** Time series datasets. **(B)** Document image datasets. **(C)** Natural image datasets. **(D)** Medical image datasets. **(E)** Synthetic image datasets.

The continuity for time series data is defined as the sum of the absolute changes between each pair of subsequent points within the attribution map. For image attributions, continuity is computed as the sum of absolute gradients in both spatial directions of the attribution map. A smoother map results in a lower continuity. Figure 7 shows the CD diagrams for the *Continuity* across all domains. For each domain, the ranked results are averaged over all datasets and attribution methods.

The results for all domains clearly show that *Baseline* and *FedAVG* settings yield better *Continuity* scores as compared to the *DP*-based approaches. This confirms that *DP*-based private models generate significantly more discontinuous attribution maps compared to *Non-DP* training techniques. The only outlier to this observation is *EfficientNet-B4* trained on document images in Figure 7B, where surprisingly, *FedAVG-DP* achieved the highest rank. Comparing only *Baseline* and *FedAVG* models, it cannot be clearly stated whether one is better than the other, as this appears to be highly dependent on the exact model architecture and domain combination. Moreover, *FedAVG-DP* achieved better ranks as compared to *DP* in most configurations across all domains. *DP* and *Non-DP* approaches even show a clear visual separation in the CD diagram in most cases (i.e., document, natural, and medical). For document image datasets, there are only minor differences within the ranks of *DP* and *Non-DP* regions, making it very difficult to draw clear conclusions.

#### 5.3.2 Area over the perturbation curve

The *AOPC* measures how removing features deemed relevant by the explanation affects local model predictions. This provides important insights into the fidelity of the explanations. Intuitively, removing features with lower importance should affect the prediction less, whereas the deletion or perturbation of important features should result in significant prediction changes. In this experiment, features were removed sequentially starting with the most important, as per the attribution map.

**Figure 8:**
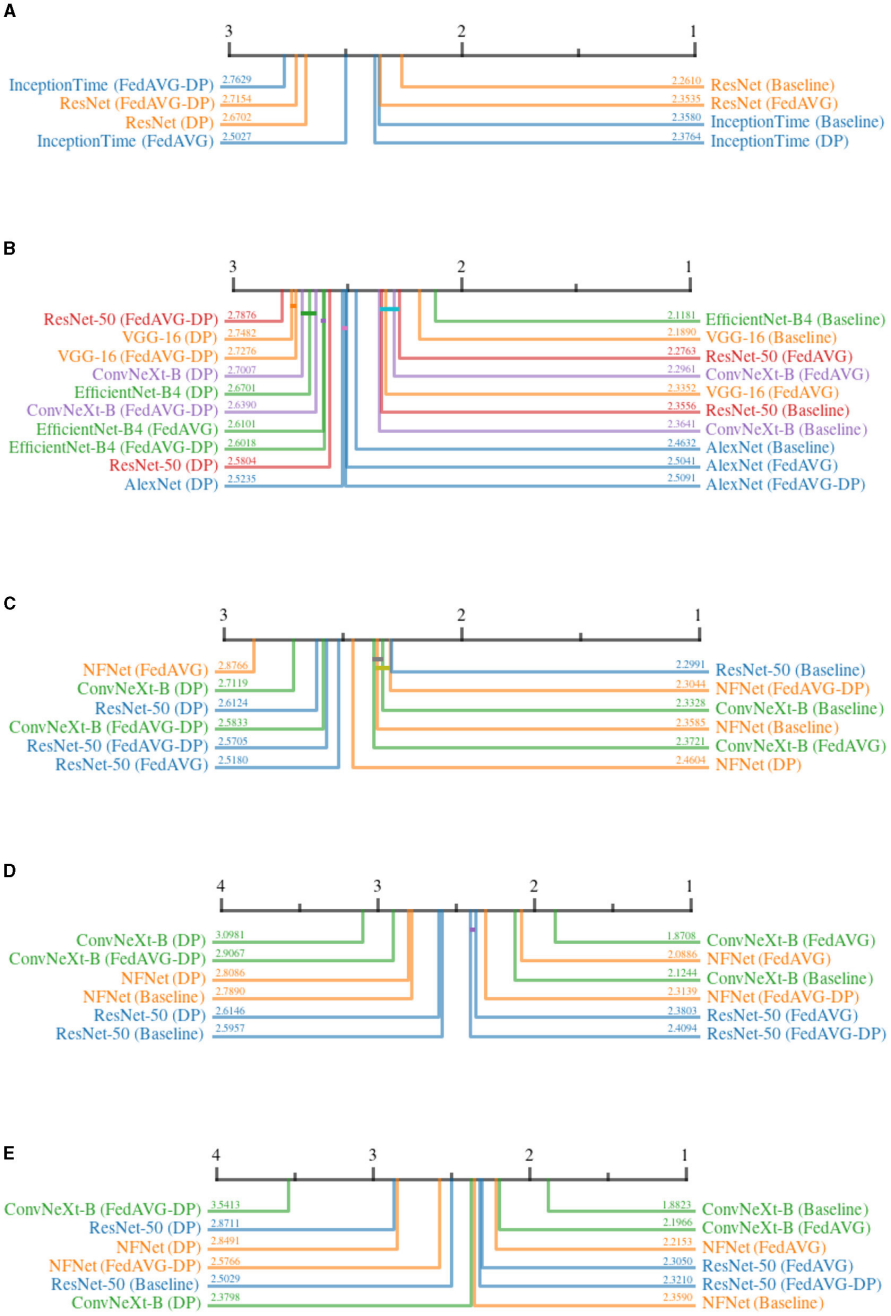
Critical difference diagrams for *AOPC*. The black bar represents the rank of each of the four individual training settings averaged over all datasets for an individual architecture. Each color represents a single architecture, and the exact rank for a particular combination is provided on the colored line. Statistical insignificance between two individual settings is indicated by a horizontal colored bar. **(A)** Time series datasets. **(B)** Document image datasets. **(C)** Natural image datasets. **(D)** Medical image datasets. **(E)** Synthetic image datasets.

Figure 8 shows all critical difference diagrams for the *AOPC* measure. Over all domains, the most prevalent pattern is that of *Non-DP*-based settings occupying the higher ranks. In the time series domain, *ResNet-50* shows the clear superiority of *Baseline* and *FedAVG* compared to *DP* and *FedAVG-DP*. However, the results from the image domain indicate that a clear superiority of *FedAVG* over *Baseline* cannot be reported. In contrast to other domains, *FedAVG*-based approaches on average achieved higher ranks on medical and synthetic images. For *InceptionTime, DP* surprisingly achieved almost similar performance as compared to *Baseline*. Apart from this outlier, *DP* almost exclusively ranked last in direct comparison with all other training settings. The presented results suggest that adding *Differential Privacy* during training decreases the explanation's fidelity.

#### 5.3.3 Infidelity

The *Infidelity* measure provides information about an explanation's fidelity by evaluating a model's adversarial robustness in regions of varying explanation relevance. Perturbations are both applied to the attribution map and the input image, while comparing the predictions of the unperturbed and noisy input. It is expected that the perturbation of a more important feature leads to a larger change in prediction.

**Figure 9:**
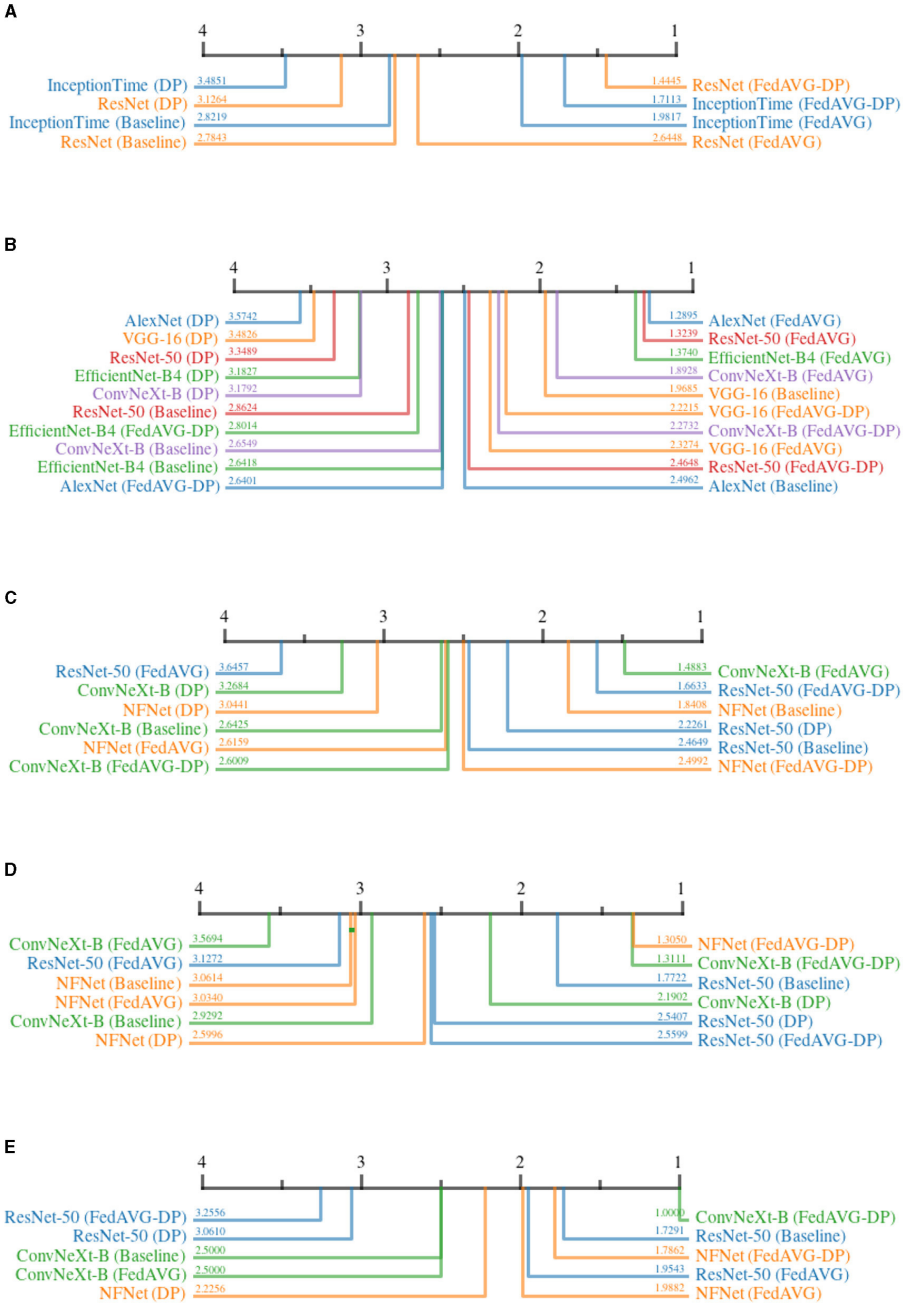
Critical difference diagrams for *Infidelity*. The black bar represents the rank of each of the four individual training settings averaged over all datasets for an individual architecture. Each color represents a single architecture, and the exact rank for a particular combination is provided on the colored line. Statistical insignificance between two individual settings is indicated by a horizontal colored bar. **(A)** Time series datasets. **(B)** Document image datasets. **(C)** Natural image datasets. **(D)** Medical image datasets. **(E)** Synthetic image datasets.

Figure 9 shows the *Infidelity* ranks for all configurations. In the time series domain, approaches involving *FedAVG* achieved the highest scores. Interestingly, the addition of *DP* to *FedAVG* settings resulted in higher scores, whereas the sole use of *DP* during training led to the worst outcomes. For all image domains, again, results depend on the architecture and the domain. However, there is an overall tendency similar to the results of the time series domain, with *DP* being the worst. Furthermore, *FedAVG* approaches being the best performing approach for time series datasets. The *Baseline* setting ranked highest in several configurations, such as *ResNet-50* in medical and synthetic images, and *NFNet* in natural images. This indicates that *FedAVG* increases adversarial robustness and fidelity, while *DP* alone leads to lower fidelity.

#### 5.3.4 Sensitivity

In contrast to the *Infidelity, Sensitivity* quantifies the fidelity by perturbing the input directly. The change in the generated explanation is measured before and after the input is insignificantly perturbed. Small changes in the input should not result in large changes in the attribution map.

**Figure 10:**
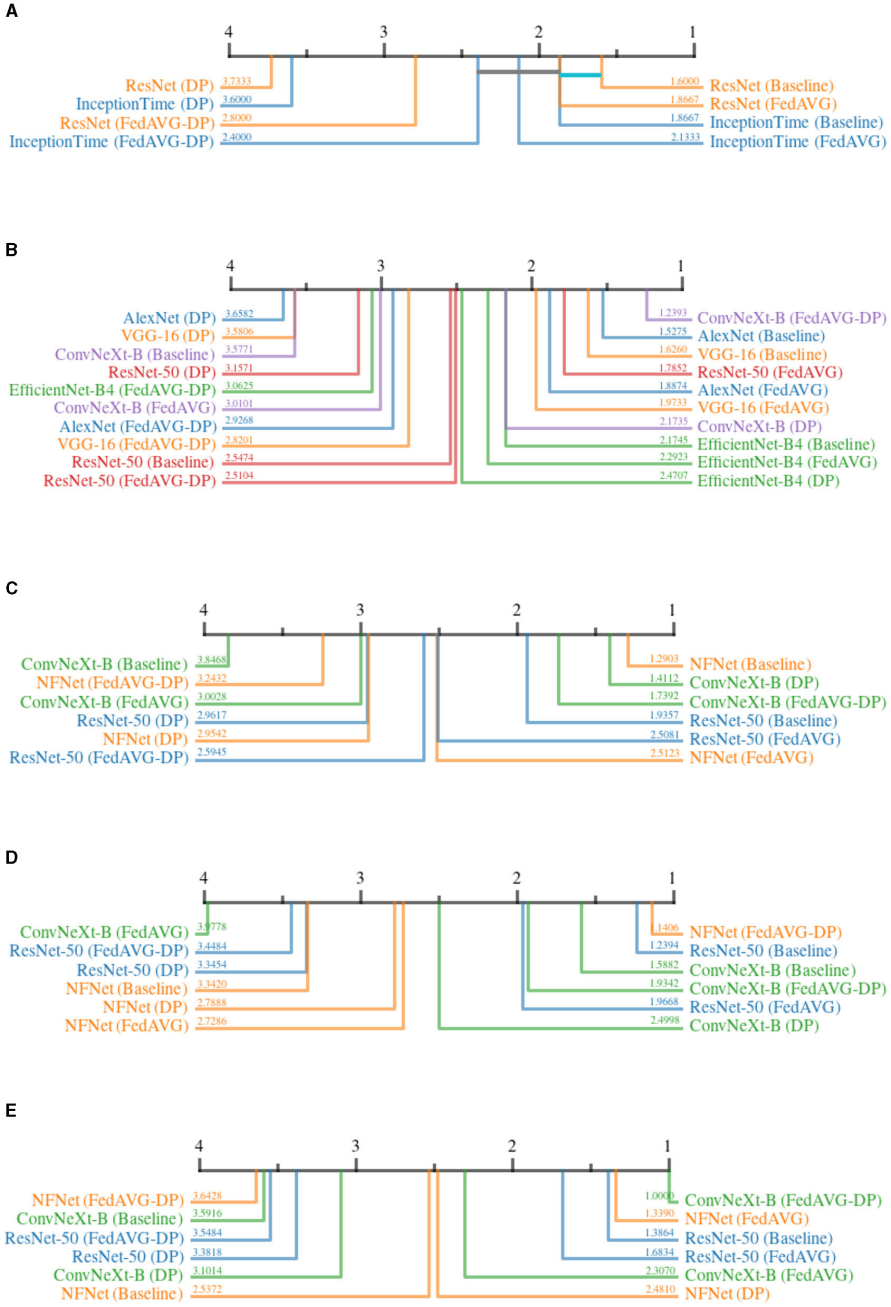
Critical difference diagrams for *Sensitivity*. The black bar represents the rank of each of the four individual training settings averaged over all datasets for an individual architecture. Each color represents a single architecture, and the exact rank for a particular combination is provided on the colored line. Statistical insignificance between two individual settings is indicated by a horizontal colored bar. **(A)** Time series datasets. **(B)** Document image datasets. **(C)** Natural image datasets. **(D)** Medical image datasets. **(E)** Synthetic image datasets.

Figure 10 shows the *Sensitivity* ranks for all configurations. For the time series domain, Figure 10A shows a clear ranking with *Baseline* and *FedAVG* being superior to *FedAVG-DP*, followed by *DP*. However, no clear statistical distinction can be made between *Baseline, FedAVG*, and *FedAVG-DP* for *InceptionTime*, and for *Baseline* and *FedAVG* for *ResNet-50*. The superiority of *Non-DP*-based over *DP*-based approaches is further confirmed by seven more configurations within the different image domains, including *ResNet-50* in natural, medical, and synthetic images. Interestingly, both *DP*-based methods ranked highest in combination with *ConvNeXt* in all image-domains except for medical imaging.

#### 5.3.5 Ground truth concordance

Measuring the concordance of attribution maps and ground truth explanations is the best way to ensure the truthfulness of explanations, but comes with some limitations. *Ground Truth Concordance* can only be computed on synthetically constructed datasets without ambiguous decision paths. Therefore, the *SCDB* dataset was utilized, which provides segmentation maps for the different visible shapes to construct ground truth explanation maps containing only decision-relevant shapes for each image.

**Figure 11:**
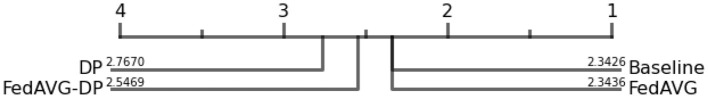
Critical difference diagram for *Ground Truth Concordance* on the SCDB dataset. The black bar represents the rank of each of the four individual training settings averaged over all architectures. The exact rank for a combination is provided on the line pointing to the setting. *FedAVG* and *Baseline* settings clearly outperform *DP*-based techniques.

Figure 11 shows the critical difference diagram for the *Ground Truth Concordance* computed over all attribution methods for the *SCDB* dataset. The ranking clearly indicates the superiority of *Baseline* and *FedAVG* settings over *DP*-based training techniques. Moreover, it can be observed that the addition of *FedAVG* to the *DP*-trained setting alleviates the divergence from the ground truth explanations. Overall, it can be noted that *DP*-based methods indeed reduce the fidelity of models, while there is promising evidence that a *DP*-based training in a federated constellation can mitigate its effects to a certain degree.

### 5.4 Impact of noise on different settings

The results so far suggested that the introduction of *DP* during the training process has a considerable impact on the generated explanations. Moreover, it was found that using the combination of *FedAVG* and *DP* can sometimes mitigate the negative effects of the added noise during the training process. This section covers an investigation whether the degree to which the quality of explanations is affected, differs, for different attribution methods and datasets. Therefore, the relative increase in continuity score was measured when comparing the *Baseline* with the *DP* training setting. A higher relative increase indicates a bigger impact, resulting in a lower rank.

#### 5.4.1 Impact of noise on different attribution methods

**Figure 12:**
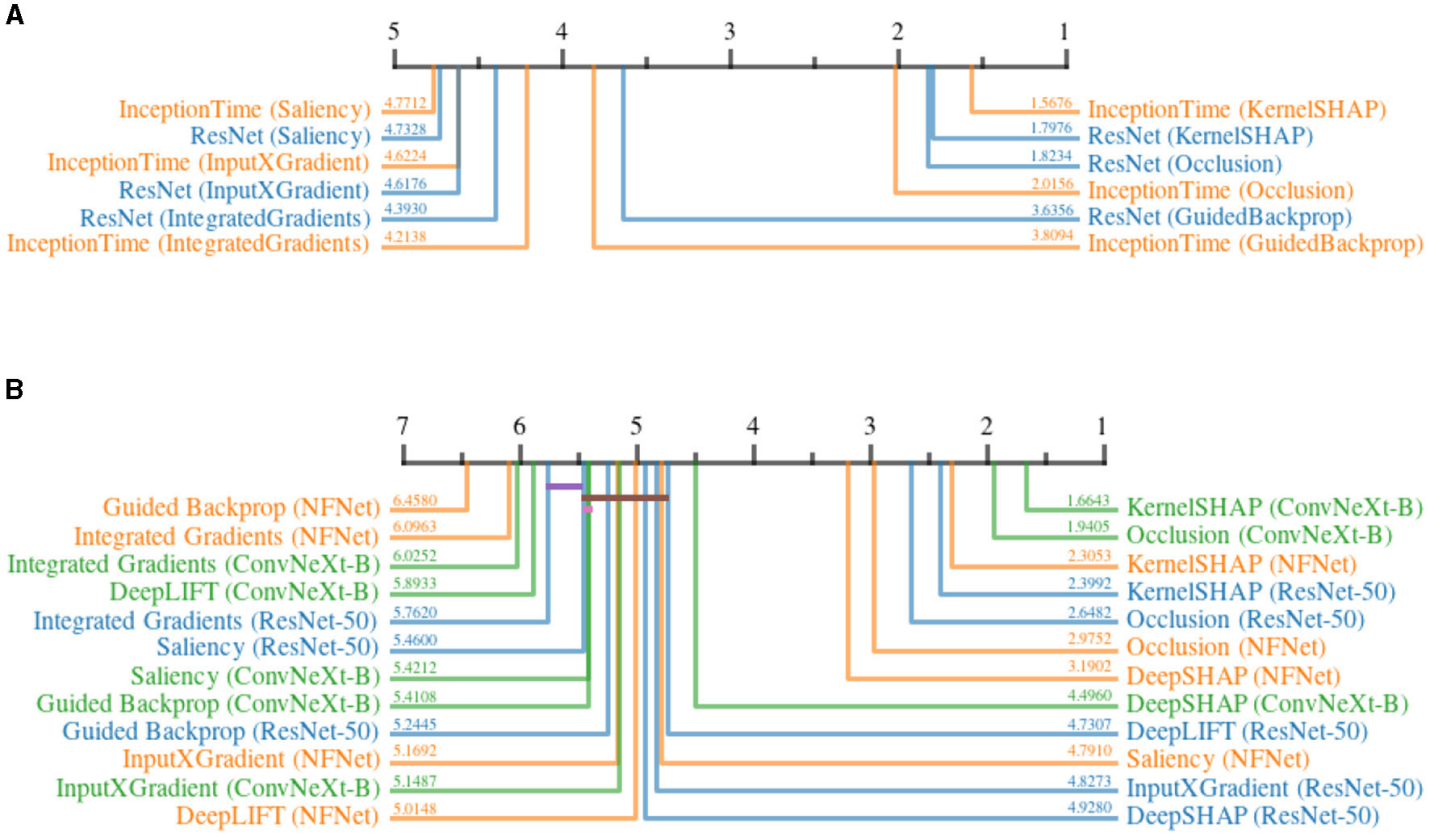
Critical difference diagrams, showing the impact of *Differential Privacy* on the quality of explanations generated by different attribution methods. The quality is measured in terms of relative increase in continuity before and after adding *DP*. The black bar represents the rank of each attribution method averaged over all datasets for an individual architecture. Each color represents a single architecture, and the exact rank for a particular combination is provided on the colored line. Statistical insignificance between two individual methods is indicated by a horizontal colored bar. **(A)** Time series datasets. **(B)** image datasets.

Figure 12 shows the ranks of different attribution methods when applied to different architectures before and after adding *DP* to the training, for the time series and image datasets. For both modalities, a prominent separation of two distinct groups can be noticed. In time series datasets, both *KernelSHAP* and *Occlusion* are affected significantly less by differential privacy as compared to the remaining, gradient-based methods. Within the gradient-based methods, *GuidedBackpropagation* suffered less from *DP*, followed by *IntegratedGradients, InputXGradient*, and *Saliency*. The results do not indicate a clear advantage of using one model architecture over the other. Similarly, *KernelSHAP* and *Occlusion* clearly outperformed most other methods when applied to the image datasets. *DeepSHAP* is the only exception, achieving a similar score to *Occlusion* when applied to *NFNet*. By approximating SHAP values using gradients (Lundberg and Lee, 2017), *DeepSHAP* achieved higher robustness compared to other gradient-based methods for two out of three models. For *ResNet-50, InputXGradient* and *DeepLIFT* slightly outperformed *DeepSHAP*. Among all gradient-based methods, *IntegratedGradients* scored last for most of the model architectures, whereas the other methods do not paint a clear picture. Similar to the time-series results, the values do not indicate the superiority of one model architecture over another. Overall, the results clearly show that the selection of the attribution method has a significant impact on the robustness of the generated explanations under the influence of noise introduced by *DP*.

#### 5.4.2 Impact of noise on different datasets

**Figure 13:**
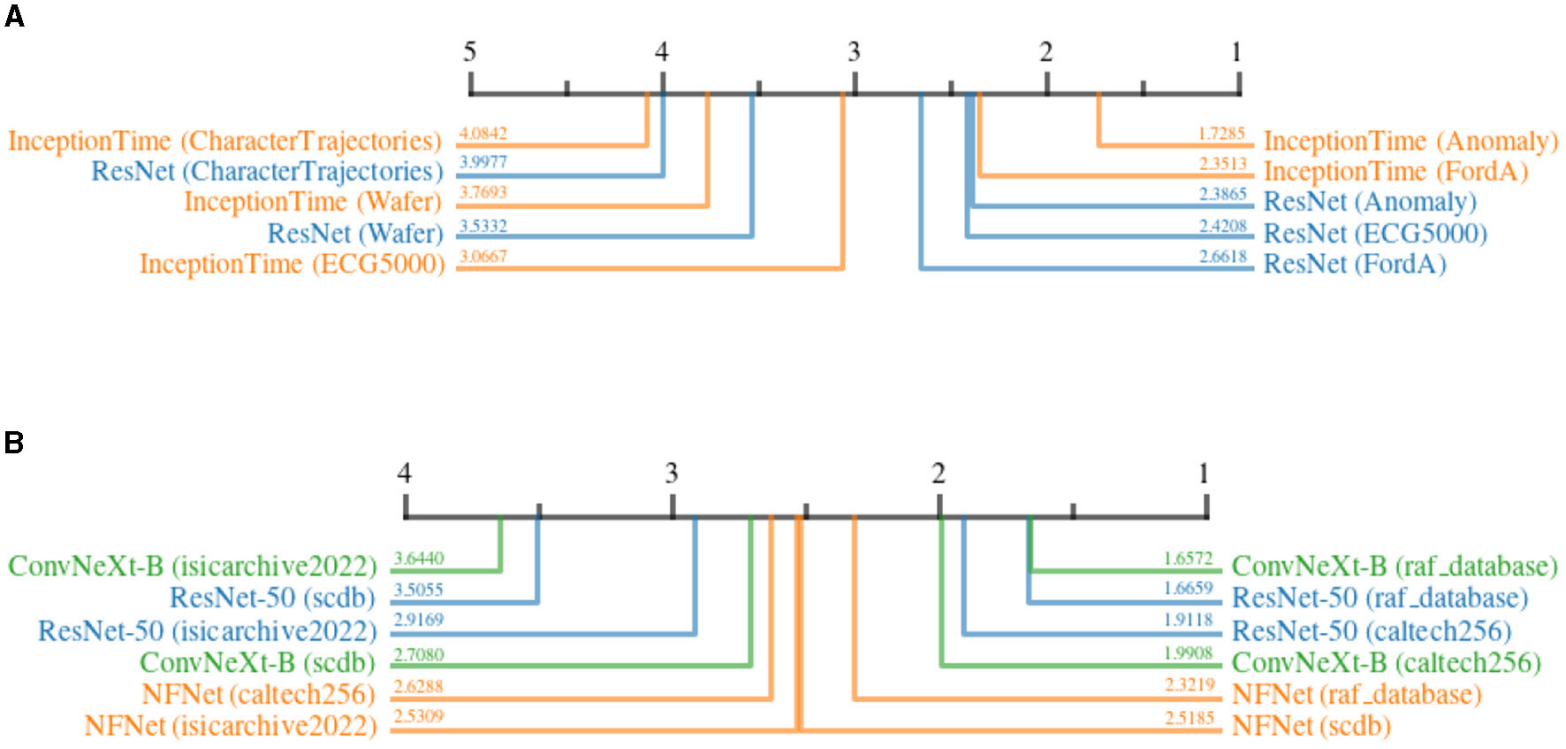
Critical difference diagrams, showing the impact of *Differential Privacy* on the quality of explanations when applied to different datasets. The quality is measured in terms of relative increase in continuity before and after adding *DP*. The black bar represents the rank of the dataset averaged over all attribution methods for an individual architecture. Each color represents a single architecture, and the exact rank for a particular combination is provided on the colored line. **(A)** Time series datasets. **(B)** image datasets.

Figure 13 shows the impact of *DP* on the quality of explanations for different datasets. For the time series domain, it can be seen that noise has the least impact on the *Anomaly* dataset, as the decision-relevant anomaly is not affected much by the added noise. On the other hand, *Character Trajectories* dataset is highly affected by noise. This can be explained by the fact that the dataset consists of raw sensor values that describe drawn letters. Slight noise distributed over the time series can have a devastating influence on the meaning of a given sample, as the error adds up over time. In the image-domain, *RAF-Database* and *Caltech-256* are influenced less by noise, whereas *ISIC*, on average, shows a higher susceptibility. This is understandable, as *ISIC* heavily relies on fine-grained patterns and complex features, which might be more susceptible to added noise as compared to coarse-grained features used for emotion recognition and object detection. Surprisingly, the results suggest a rather high impact on *SCDB* as well. At first, this might seem unexpected due to the relevance of clean and uniform shapes for classification. However, considering the low resolution of input images, these shapes might be particularly fragile under the influence of noise, as small perturbations can easily shift the resemblance of one shape to another.

## 6 Discussion

In the last few years, explainability and data privacy are drastically gaining importance in the field of Deep Learning. It is therefore all the more important to take a closer look at their interaction. The presented results revealed a significant impact of privacy-preserving training techniques on generated explanations. However, the influence on XAI strongly depends on the privacy technique used, as well as other factors.

### 6.1 The disparate impact of PPML on model convergence

First, it has been shown that not every PPML method has the same impact on model performance. *DP*-based models were shown to almost always deteriorate the test accuracy. Moreover, experience showed that they drastically complicate model convergence and hyperparameter search. *FedAVG*, on the other hand, yielded accuracies similar to the *Baseline* setting, sometimes even improving the results. It has to be mentioned, though, that both *DP* and *FedAVG* follow different goals in the domain of privacy. Whereas *DP* aims at preventing models to capturing individual sample information, which could be used for reconstruction, *FedAVG* mainly aims at minimizing the exposure of sensitive information by keeping the training data local. Although *FedAVG* also generates an aggregated model which might have less vulnerability to reconstruction attacks due to averaging effects, it still needs to transfer information about the local models to the orchestration server. Therefore, the combination of *FedAVG* and *DP* provide the highest privacy, often yielding similar performance compared to only *DP*.

### 6.2 Differential privacy leads to noisy attribution

The qualitative and quantitative analysis revealed various interesting findings regarding the impact of different privacy-preserving techniques on explanations. *Differential Privacy*, for example, stood out in almost all configurations for its property to add noise to the attribution maps. This has been reported in many individual samples and could be confirmed by dataset level analysis, as well as quantitative analysis, where *DP*-based methods stood out for increased *Continuity* values. One possible reason for this phenomenon is the addition of noise during the training process with *DP*. The introduction of noise in the parameter update most likely leads to contortions in the parameter space, which are never completely compensated, and translate into the prediction process. This effect might be counteracted by slightly tweaking the optimization, such as fine-tuning public datasets, or by increasing the batch sizes during training.

### 6.3 Perturbation-based attribution is more robust

The degree to which noise is added has been investigated in Section 5.4. The results suggest, that perturbation-based methods are a lot less prone to changing their explanation's *Continuity* under influence of noise. Inspecting the individual examples, as well as the average heatmaps in Figure 6, this finding can again be verified. The difference between *Occlusion* and *Saliency* is particularly notable in the contrast between areas of higher and lower relevance. Whereas *Saliency* produces monotonous heatmaps, peaks and areas of interest are much more prominently highlighted in the average *Occlusion* maps. The main reason that investigated perturbation-based methods are less affected by noise in terms of *Continuity* is that they aggregate relevance over patches, instead of processing relevance pixel-wise. By neglecting fine nuances in the relevance, the randomly introduced noise is likely canceled out within a patch. However, *Continuity* is only a mathematical approximation of an explanation's interpretability. Figures 2–4 illustrate that *Occlusion*-based explanations are often significantly changed when introducing *DP* during training. Furthermore, the high interpretability of heatmaps is worthless if their fidelity is not ensured. As reported in Section 5.3, *DP* exclusively led to the deterioration of metrics indicating an explanation method's fidelity. Therefore, even when applying *Occlusion*, it needs to be clarified how truthful the generated explanations remain to be.

### 6.4 Federated learning can improve attributions

In contrast to *DP, Federated Learning* often resulted in smoother attribution. Interestingly, combining *FedAVG* with *DP* often times even led to more continuous attribution maps compared to the *Baseline* setting, reducing the negative effects introduced by *DP* alone. However, *FedAVG-DP* has also been reported to decrease the fidelity of explanations in many cases. Therefore, whenever XAI is required and *Differential Privacy* is applied, it might be worth considering a combination of *DP* and *Federated Learning*. This will also be possible in cases where *Federated Learning* is not required, as the federated setting can easily be simulated by dividing the dataset into chunks. Although some outlier experiments report a better *Continuity* score for *Baseline* settings, the fact that *FedAVG* leads to better *Continuity* scores has a strong theoretical basis. Averaging models during training inevitably prevents the final model from overemphasizing granular features or noise.

### 6.5 The importance of task granularity

The present study also indicated that the influence of PPML on XAI is not really dependent on the application domain, but rather on the choice and feature scales of the dataset at hand. The noise introduced by *DP* has, above all, a detrimental impact on classification tasks that rely on fine-grained and nuanced features or patterns. Simpler anomaly detection tasks or tasks focusing on the detection of overall, coherent structures seem less affected by privacy-preserving training techniques.

### 6.6 XAI as a privacy threat

Besides the different influences PPML has on XAI, there is another fact that needs to be considered when combining both techniques. No matter how private a system has been made, exposing an explanation is in itself always a potential point of attack for a system, revealing sensitive information about the decision-making process. This is, for instance, particularly evident with *Saliency*, which provides the raw gradients of a single input instance. For truly critical applications, explanations should only be issued to a small group of authorized users and only if really required. Moreover, it might even be necessary to further obfuscate the exact generation process of explanations for applications with extremely high privacy requirements.

### 6.7 Limitations

This study revealed several general trends which will affect explanations on a global scale when applying private training strategies to DL-based models. However, one major limitation of such studies is the examined basis of comparison. When comparing explanations of separate model instances, there is always the risk of obtaining different local minima, i.e., different classification strategies. Previous research (Ilyas et al., 2019) suggests that one dataset can have multiple, redundant, but fundamentally different features. Therefore, even models with identical test performance could have, in theory, picked up entirely different cues to solve the same problem, hence yielding deviant explanations per model. When training models using different training strategies, it cannot be avoided to obtain models with deviating classification strategies. This is also clearly reflected in the naturally lower model performance of *DP*-based models.

Further limitations are related to the evaluation of the explanation's quality through quantitative metrics. As already mentioned, quantitative quality metrics for XAI are simply mathematical approximations of factors that could account for human interpretability or test assumptions of fidelity that should be satisfied by good explanations. Many such metrics still have inherent limitations like *AOPC, Sensitivity*, and *Infidelity*, introducing out-of-distribution samples through the perturbation of samples. *Ground Truth Concordance* assumes that, for each sample, there is exclusively one single decision path, and therefore a ground truth explanation. To approach this assumption, a synthetic dataset was utilized that allowed the construction of ground truth segmentation maps, highlighting all decision-relevant shapes. For somewhat complicated problems, explanations are always redundant, as are the corresponding human problem definitions. The human-made logic behind the dataset postulates that a set of pre-defined shapes (i.e., star or triangle) need to be present to associate a sample with a class. However, instead of selecting the entirety of a shape, networks could also simply define triangles by the angle of their apexes. This way, the explanation would not need to cover the complete shape, but only an arbitrarily small area around a single apex.

## 7 Conclusion

Both eXplainable AI and privacy-preserving machine learning constitute pivotal technologies for the safe translation of state-of-the-art AI algorithms into everyday applications. It is particularly important to get an early understanding of the effect of private training on XAI, to actively develop countermeasures, and avoid blind interpretations of explanations. This work showed that, although the exact effect on explanations depends on a multitude of factors including the privacy technique, dataset, model architecture, and XAI method, some overall trends can be identified. It has been found that *Differential Privacy*, on average, decreases both the *Interpretability* and *Fidelity* of heatmaps. However, *Federated Learning* was found to moderate both effects when used in combination. When used alone, *FedAVG* was even found to sometimes improve the *Interpretability* of attribution maps by generating more continuous heatmaps. The results suggest considering *Federated Learning* before *Differential Privacy*, where appropriate. Moreover, it is recommended always to choose *Differential Private Federated Learning* as well as perturbation-based XAI methods, if an application requires both privacy and explainability. As the first work to investigate the impact of privacy on XAI, this study opens up a series of interesting follow-up questions, including the in-depth analysis of the trade-off between privacy and interpretability under different privacy constraints, and the impact of using privacy techniques beyond *DP* and *FedAVG*. Moreover, the field would benefit from a more profound analysis of the effect of privacy on the interpretation by human users through application-grounded and human-grounded evaluation methods. The union of PPML and XAI solves one of the remaining regulatory and safety-critical hurdles, freeing the way for innovative and high-performing AI-based applications that can bring significant advancements in crucial domains of our everyday lives.

## Data availability statement

The datasets analysed during the current study are available in the following repositories: Anomaly Detection, Character Trajectories, ECG5000, FordA, and Wafer can be accessed via the UEA & UCR repository (https://timeseriesclassification.com/). The RAF-Database can be accessed via the official website (http://www.whdeng.cn/raf/model1.html). Caltech-256 can be accessed via Kaggle (https://www.kaggle.com/datasets/jessicali9530/caltech256). ISIC can be accessed via the website of the International Skin Imaging Collaboration (ISIC) (https://www.isic-archive.com). SCDB can be accessed via the original github repository (https://github.com/adrianolucieri/SCDB). RVL-CDIP can be accessed via the official project page (https://adamharley.com/rvl-cdip/). Tobacco3482 can be accessed via Kaggle (https://www.kaggle.com/datasets/patrickaudriaz/tobacco3482jpg).

## Author contributions

Data collection, analysis, and the first draft of the manuscript were written by SS, DM, and AL. All authors commented on previous versions of the manuscript, contributed to the study conception and design, and read and approved the final manuscript.

## Footnotes

^1^Accesible under https://timeseriesclassification.com/.

^2^The ISIC Archive is accessible at https://www.isic-archive.com.

^3^ https://www.kaggle.com/patrickaudriaz/tobacco3482jpg
