# Supplementary material for: The privacy-explainability trade-off: unraveling the impacts of differential privacy and federated learning on attribution methods
Source: Front Artif Intell. 2024 Jul 3;7:1236947. doi: 10.3389/frai.2024.1236947 (PMC11253022; doi:10.3389/frai.2024.1236947)
Supplement: Supplementary file 1 [file Data_Sheet_1.pdf]

1 Additional Results

1.1 Analysis of Individual Sample Attributions

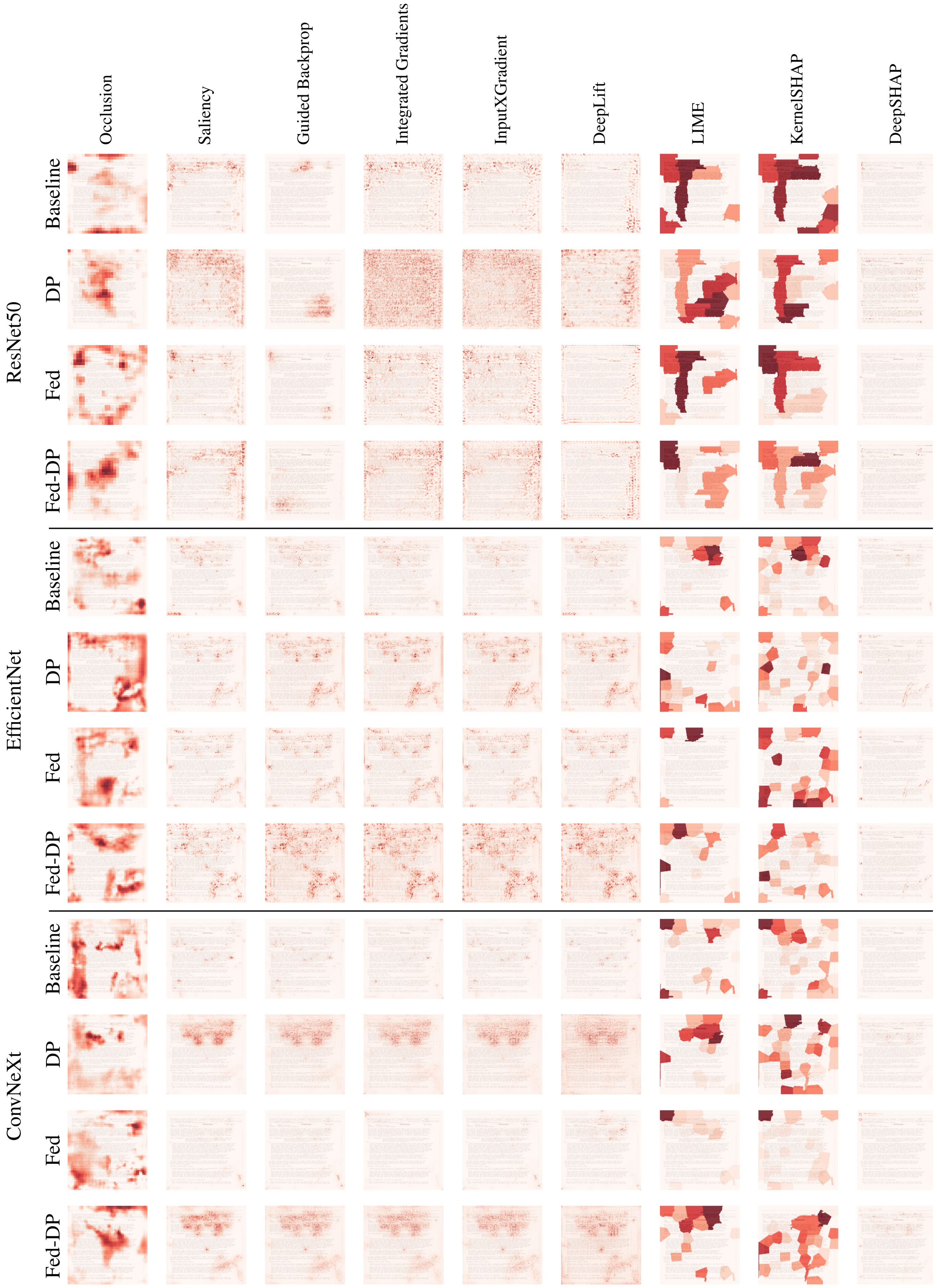

Figure 1: Examples of attribution maps of a single, randomly selected and correctly classified sample from the *RVL-CDIP* dataset. All attribution maps have been superimposed over the grayscale sample, for easier interpretation. Red regions indicate positive attribution to the predicted class.

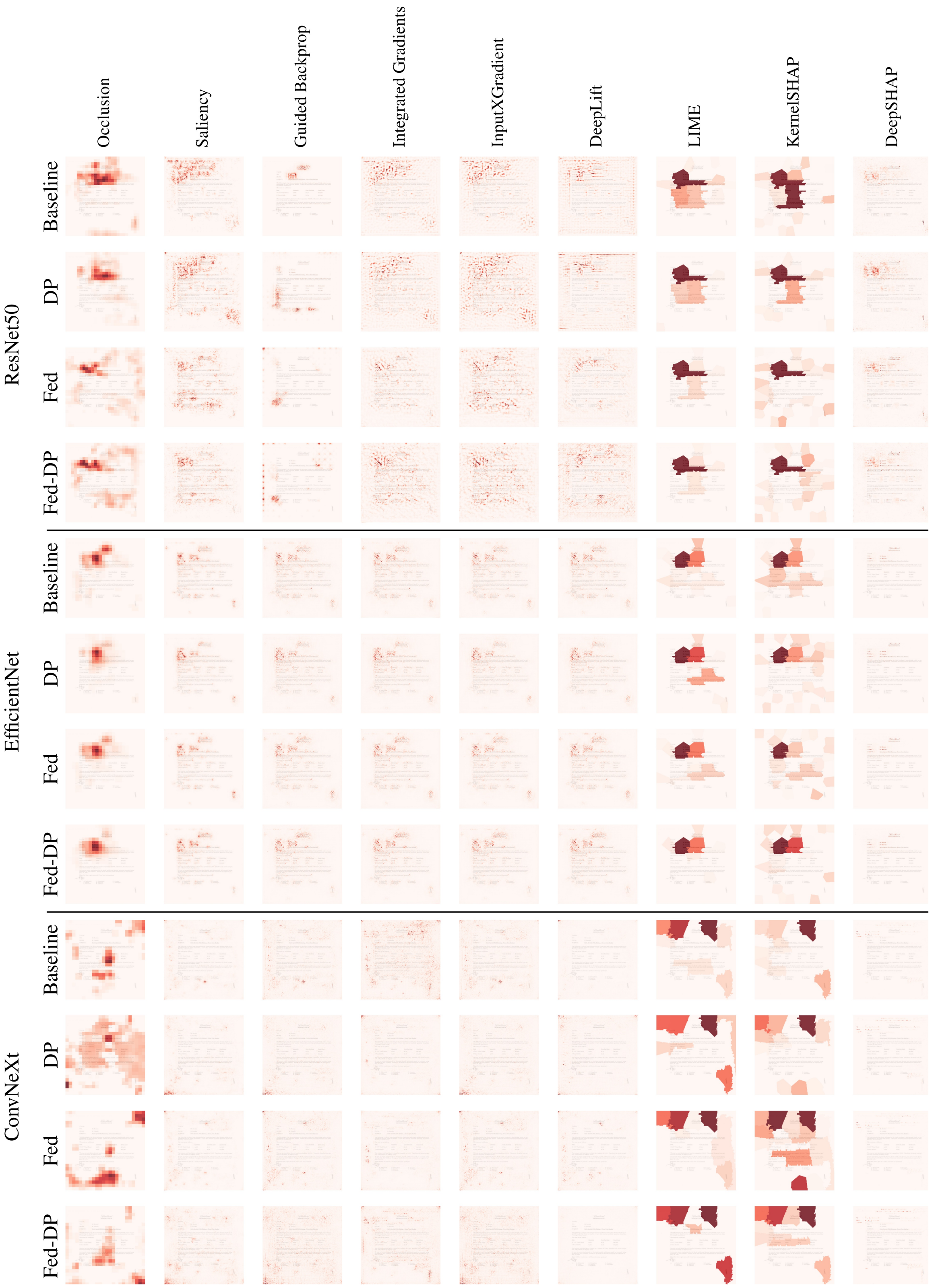

Figure 2: Examples of attribution maps of a single, randomly selected and correctly classified sample from the *Tobacco3482* dataset. All attribution maps have been superimposed over the grayscale sample, for easier interpretation. Red regions indicate positive attribution to the predicted class.

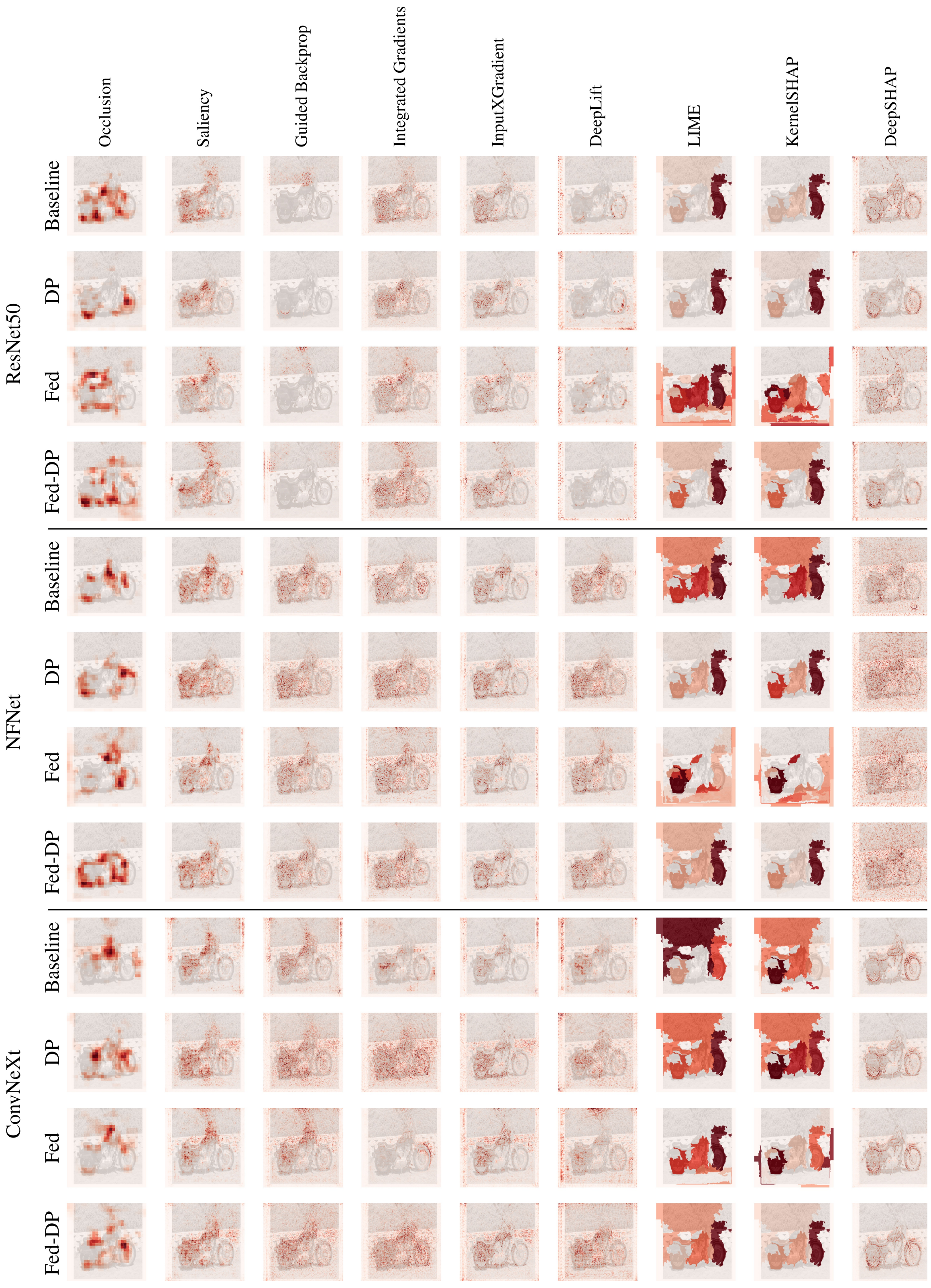

Figure 3: Examples of attribution maps of a single, randomly selected and correctly classified sample from the *Caltech256* dataset. All attribution maps have been superimposed over the grayscale sample, for easier interpretation. Red regions indicate positive attribution to the predicted class.

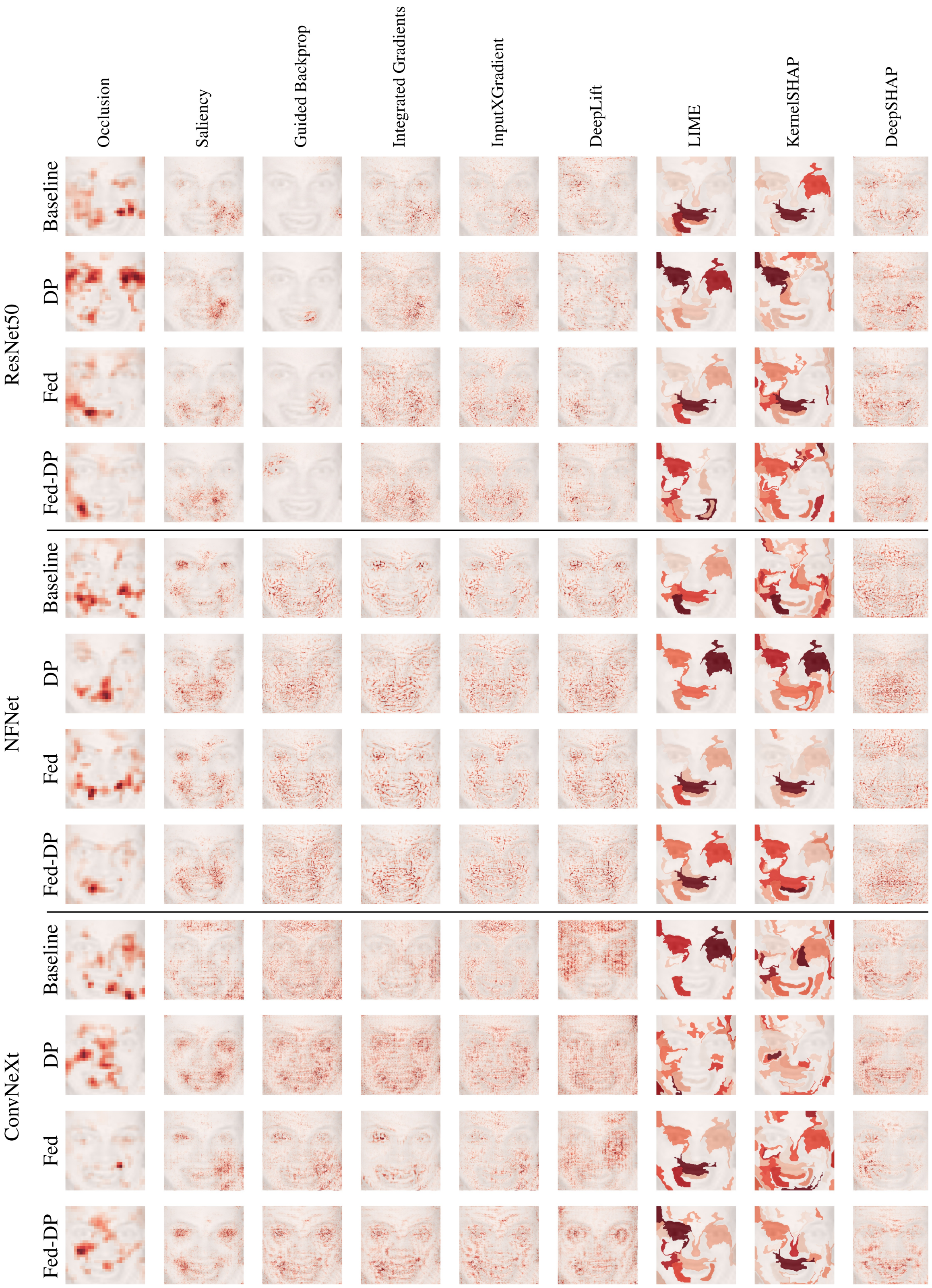

Figure 4: Examples of attribution maps of a single, randomly selected and correctly classified sample from the *RAF-Database* dataset. All attribution maps have been superimposed over the grayscale sample, for easier interpretation. Red regions indicate positive attribution to the predicted class.

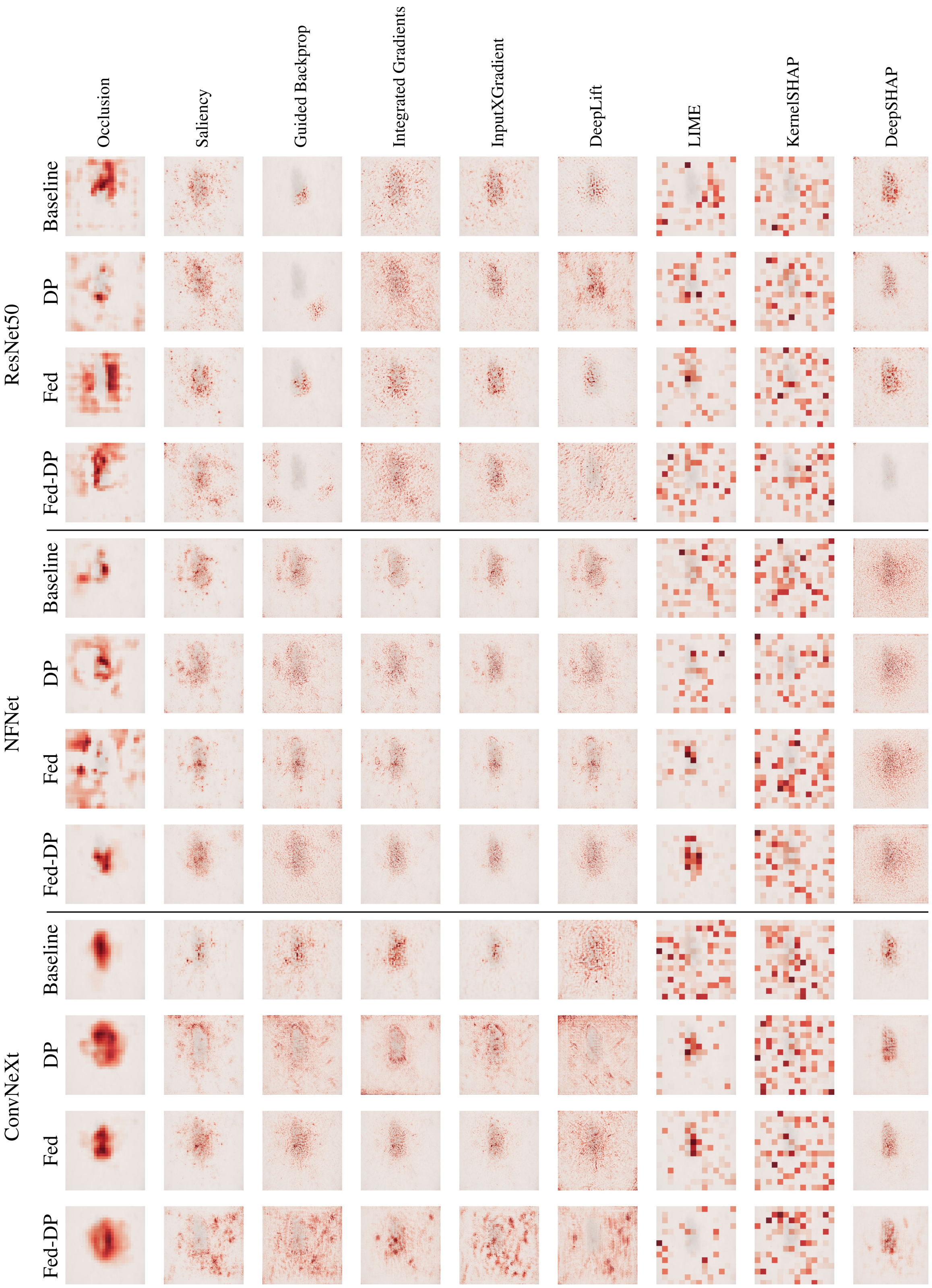

Figure 5: Examples of attribution maps of a single, randomly selected and correctly classified sample from the *ISIC* dataset. All attribution maps have been superimposed over the grayscale sample, for easier interpretation. Red regions indicate positive attribution to the predicted class.

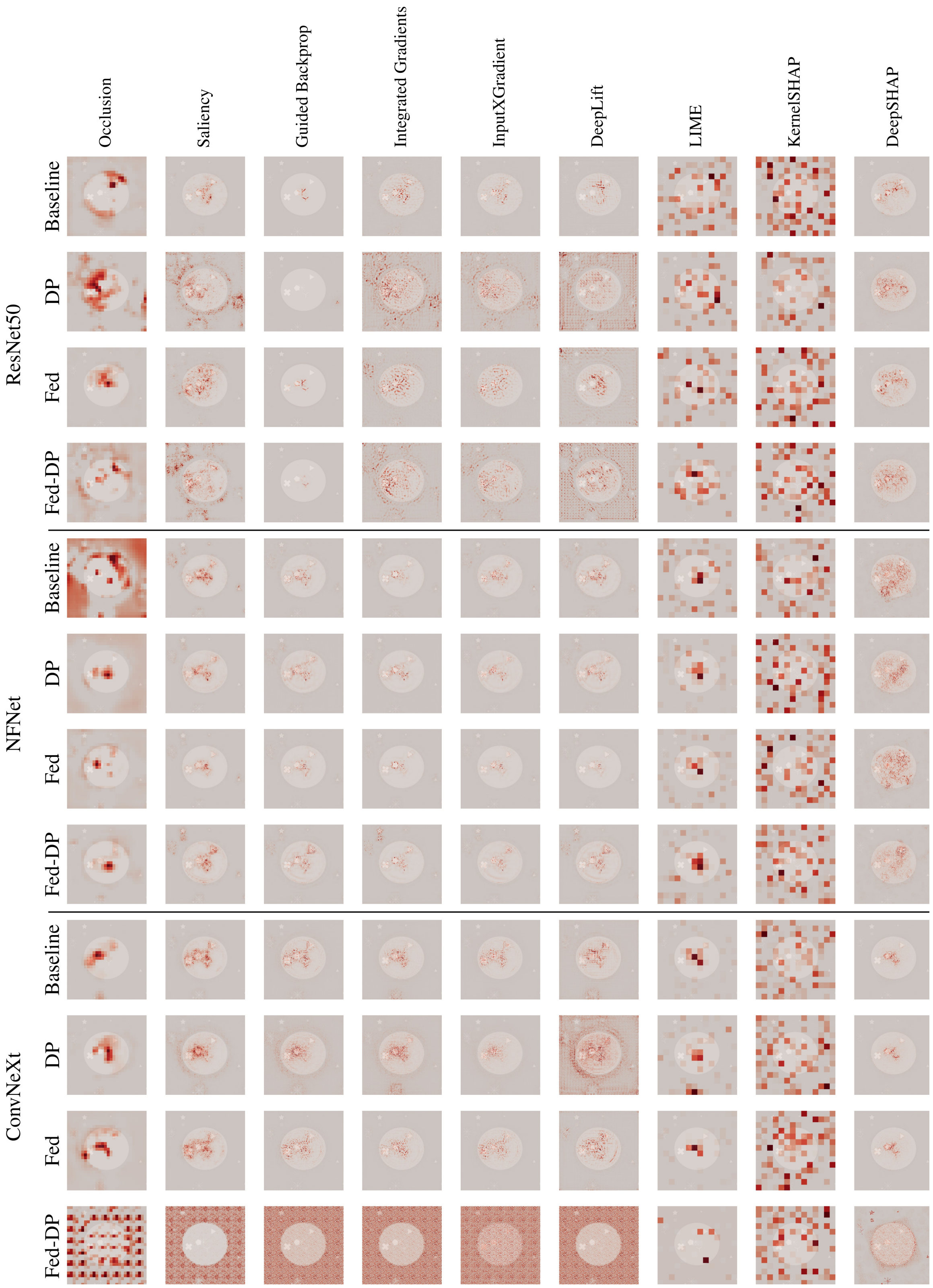

Figure 6: Examples of attribution maps of a single, randomly selected and correctly classified sample from the *SCDB* dataset. All attribution maps have been superimposed over the grayscale sample, for easier interpretation. Red regions indicate positive attribution to the predicted class.

1.2 Dataset-wide Analysis

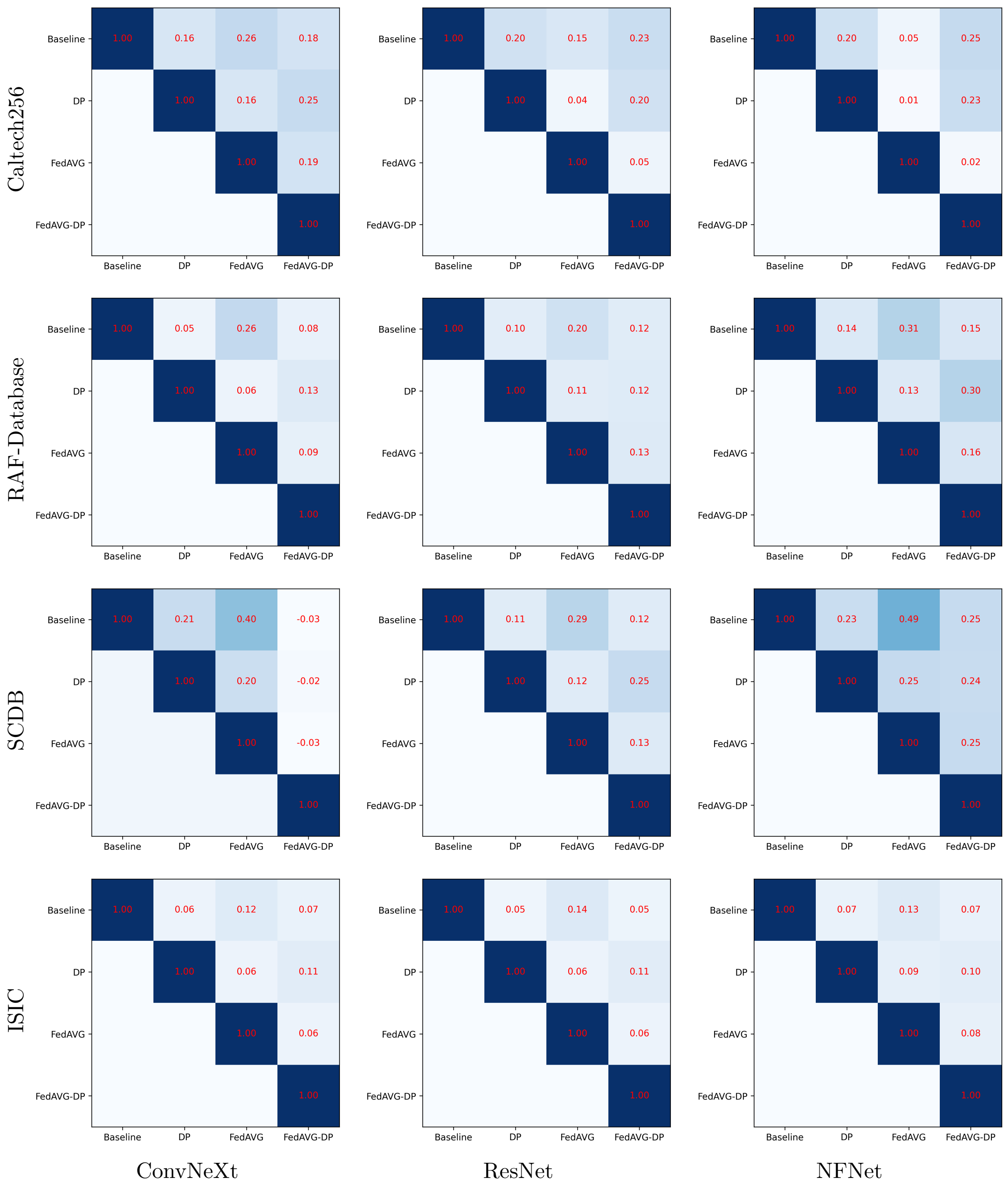

Figure 7: Shows the average Pearson correlation of the attribution maps compared between the different privacy approaches for the Caltech256, RAF-Database, ISIC, and SCDB datasets. *FedAVG* shows a higher similarity to the *Baseline* setting for most scenarios, as compared to the *DP*-based approaches.

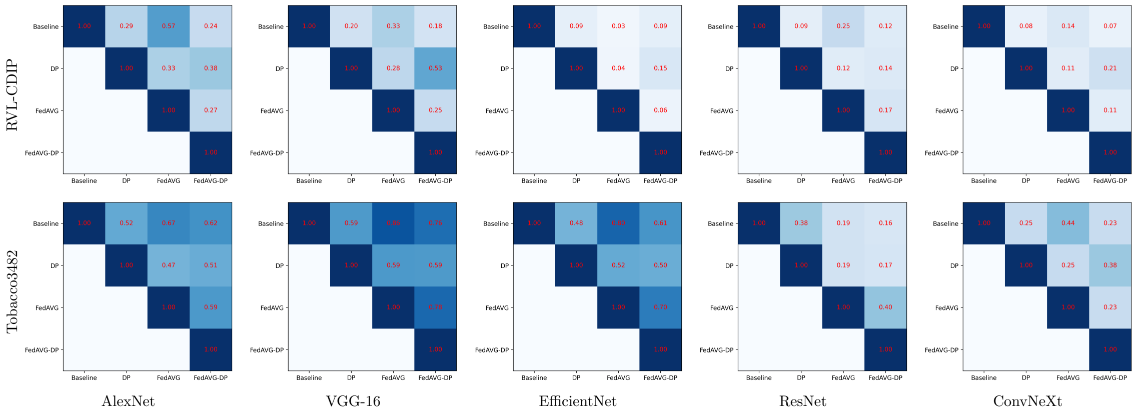

Figure 8: Shows the average Pearson correlation of the attribution maps compared between the different privacy approaches for the Tobacco3482 and RVL-CDIP datasets. *FedAVG* shows a higher similarity to the *Baseline* setting for most scenarios, as compared to the *DP*-based approaches.

1.3 PPML Algorithms

1.3.1 DP-SGD/Adam

The pseudocodes for both DP-SGD and DP-Adam algorithms are provided in Algorithm 1.

Algorithm 1 DP-SGD/Adam

Input: $\mathcal{L}(\theta) = \frac{1}{B}\sum_i \mathcal{L}(\theta, x_i)$, Dataset $\mathcal{D} = (x_1, y_1), \ldots, (x_N, y_N)$, learning rate η, gradient clipping norm C, noise scale σ, sampling rate q, target (ε, δ), privacy accountant $\mathcal{M}$, total training steps T

```
for each step t = 1, ..., T do
    B ← (sample a batch of size B with sampling probability q)
    foreach x_i ∈ B do
        // Compute gradient
        g(x_i) ← ∇_{θ_t} L(θ_t, x_i)
        // Clip gradient
        ḡ(x_i) ← g(x_i)/max(1, ||ḡ(x_i)||_2 / C)
    end
    // Add noise
    g̃ ← (1/B)(Σ_i ḡ(x_i) + N(0, σ^2 C^2 I)
    if Algorithm is DP-SGD then
        // Call SGD Update
        θ_{t+1} ← θ_t − η g̃
    else if Algorithm is DP-Adam then
        // Call Adam Update
        m_t ← β_1 m_{t−1} + (1 − β_1) g̃
        v_t ← β_2 v_{t−1} + (1 − β_2) g̃^2
        m_t ← m_t / (1 − β_1^t)
        v_t ← v_t / (1 − β_2^t)
        θ_{t+1} ← θ_t + η m_t / (√v_t + τ)
    end
    print M.get_privacy_spent(q, σ, t, δ)
end
```

1.3.2 Federated Learning Algorithms

The pseudocodes for the FedAVG, and the FedAVG-DP algorithms are given in Algorithm 2, and Algorithm 3, respectively.

Algorithm 2 FedAVG

Input: Learning rate η, total clients N_c, clients sampling rate f_c, total federated learning rounds N_R
Server:
  for *each round* $r = 1, \ldots, N_R$ **do**
    $\mathcal{S}_r \leftarrow$ (sample a set of m clients from N_c)
    foreach *each client* $k \in \mathcal{S}$ **do**
      $\theta_{k,r} \leftarrow \text{ClientUpdate}(k, \theta_{r-1})$
    end
    $\theta_r \leftarrow \sum_{k \in \mathcal{S}_r} \frac{n_k}{n} \theta_{k,r}$
  end
ClientUpdate *(k, θ)*:
  Input: $\mathcal{L}(\theta) = \frac{1}{B} \sum_i \mathcal{L}(\theta, x_i)$, $\mathcal{D}_k$ of size $\|\mathcal{D}_k\|$
  $\mathcal{B} \leftarrow$ (sample a batch of size B)
  for *each epoch* $e = 1, \ldots, N_{local}$ **do**
    foreach $b \in \mathcal{B}$ **do**
      if *Optimizer is SGD* **then**
        // Call SGD Update
        $\theta \leftarrow \theta - \eta \nabla_\theta \mathcal{L}(\theta, b)$
      else if *Optimizer is Adam* **then**
        // Call Adam Update
        $\tilde{\mathbf{g}} \leftarrow \nabla_\theta \mathcal{L}(\theta, b)$
        $m_t \leftarrow \beta_1 m_{t-1} + (1 - \beta_1)\tilde{\mathbf{g}}$
        $v_t \leftarrow \beta_2 v_{t-1} + (1 - \beta_2)\tilde{\mathbf{g}}^2$
        $m_t \leftarrow \frac{m_t}{1-\beta_1^t}$
        $v_t \leftarrow \frac{v_t}{1-\beta_2^t}$
        $\theta \leftarrow \theta - \eta \frac{m_t}{\sqrt{v_t} + \tau}$
      end
    end
  end
return θ

Algorithm 3 FedAVG-DP

Input: Learning rate η, total clients K, clients sampling rate C, total FL rounds T
Server:
  for *each round* $t = 1, \ldots, T$ **do**
    $\mathcal{S}_t \leftarrow$ (sample a set of m clients from K)
    foreach *each client* $k \in \mathcal{S}$ **do**
      $\theta_{k,t} \leftarrow \text{ClientUpdate}(k, \theta_{t-1})$
    end
    $\theta_t \leftarrow \sum_{k \in \mathcal{S}_t} \frac{n_k}{n} \theta_{k,t}$
  end
ClientUpdate *(k, θ)*:
  Input: $\mathcal{L}(\theta) = \frac{1}{B} \sum_i \mathcal{L}(\theta, x_i)$, $\mathcal{D}_k$ of size $\|\mathcal{D}_k\|$
  $\theta \leftarrow \text{DP-SGD}(\mathcal{L}(\theta), \mathcal{D}_k)$ or $\text{DP-Adam}(\mathcal{L}(\theta), \mathcal{D}_k)$
return θ
